# Obesity-Induced Cellular Senescence Drives Anxiety and Impairs Neurogenesis

**DOI:** 10.1016/j.cmet.2018.12.008

**Published:** 2019-05-07

**Authors:** Mikolaj Ogrodnik, Yi Zhu, Larissa G.P. Langhi, Tamar Tchkonia, Patrick Krüger, Edward Fielder, Stella Victorelli, Rifqha A. Ruswhandi, Nino Giorgadze, Tamar Pirtskhalava, Oleg Podgorni, Grigori Enikolopov, Kurt O. Johnson, Ming Xu, Christine Inman, Allyson K. Palmer, Marissa Schafer, Moritz Weigl, Yuji Ikeno, Terry C. Burns, João F. Passos, Thomas von Zglinicki, James L. Kirkland, Diana Jurk

**Affiliations:** 1 Institute for Cell and Molecular Biosciences, Newcastle University Institute for Ageing, Campus for Ageing and Vitality, Newcastle upon Tyne NE4 5PL, UK; 2 Robert and Arlene Kogod Center on Aging, Mayo Clinic, 200 First Street SW, Rochester, MN 55905, USA; 3 Department of Anesthesiology, Stony Brook School of Medicine, 101 Nicolls Road, Stony Brook, New York, NY 11794, USA; 4 Center for Developmental Genetics, Stony Brook University, 100 Nicolls Road, Stony Brook, New York, NY 11794, USA; 5 Department of Nano-, Bio-, Information Technology and Cognitive Science, Moscow Institute of Physics and Technology, Moscow, Russia; 6 Cold Spring Harbor Laboratory, Cold Spring Harbor, New York, NY, USA; 7 The Barshop Institute for Longevity and Aging Studies, San Antonio, Department of Pathology, The University of Texas Health Science Center at San Antonio, Research Service, Audie L. Murphy VA Hospital (STVHCS), San Antonio, TX 78229, USA; 8 Departments of Neurologic Surgery and Neuroscience, Mayo Clinic, 200 First Street SW, Rochester, MN 55905, USA; 9 Near East University, Arts and Sciences Faculty, Molecular Biology and Genetics, Nicosia, North Cyprus POB 99138 Mersin 10, Turkey; 10 Department of Physiology and Biomedical Engineering, Mayo Clinic, Rochester, MN 55905, USA

**Keywords:** aging, senescence, obesity, anxiety, anxiety-like behavior, neurogenesis, high-fat diet, brain, stem cells, lipid droplets

## Abstract

Cellular senescence entails a stable cell-cycle arrest and a pro-inflammatory secretory phenotype, which contributes to aging and age-related diseases. Obesity is associated with increased senescent cell burden and neuropsychiatric disorders, including anxiety and depression. To investigate the role of senescence in obesity-related neuropsychiatric dysfunction, we used the INK-ATTAC mouse model, from which p16^Ink4a^-expressing senescent cells can be eliminated, and senolytic drugs dasatinib and quercetin. We found that obesity results in the accumulation of senescent glial cells in proximity to the lateral ventricle, a region in which adult neurogenesis occurs. Furthermore, senescent glial cells exhibit excessive fat deposits, a phenotype we termed “accumulation of lipids in senescence.” Clearing senescent cells from high fat-fed or leptin receptor-deficient obese mice restored neurogenesis and alleviated anxiety-related behavior. Our study provides proof-of-concept evidence that senescent cells are major contributors to obesity-induced anxiety and that senolytics are a potential new therapeutic avenue for treating neuropsychiatric disorders.

## Highlights

• Obesity drives senescence in glial cells in the LV of mouse brains
• Obesity-induced senescence drives fat deposits in PV areas of the brain
• Senescent cell clearance in obesity restores neurogenesis in the SVZ
• Senescent cell clearance alleviates obesity-induced anxiety-like behavior

## Graphical Abstract

**Figure:**
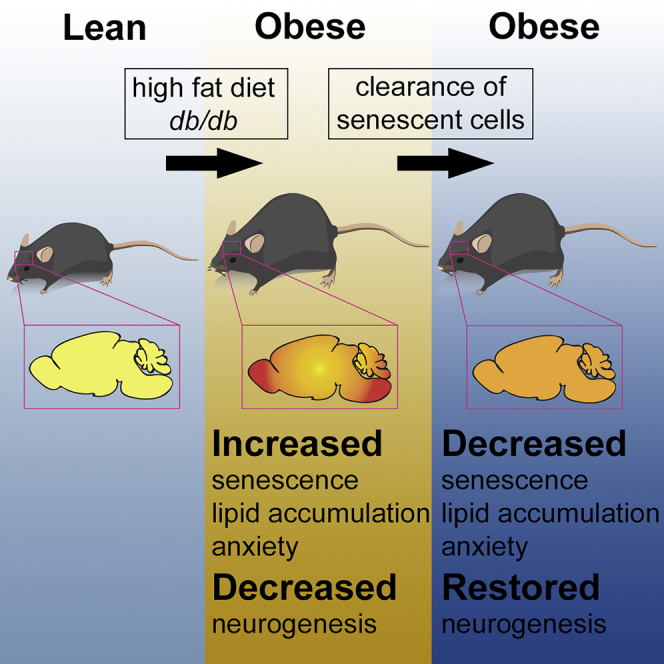

## Teaser

Obesity, a growing health problem in western societies, is associated with increased senescent cells and neuropsychiatric disorders, including anxiety and depression. Ogrodnik and colleagues found that clearance of senescent cells in obese mice alleviates anxiety. Our study provides proof-of-concept evidence that senolytics are a potential new therapeutic avenue for treating neuropsychiatric disorders.

## Introduction

Obesity is associated with a range of neurodegenerative and psychiatric disorders, including depression and anxiety (Gariepy et al., 2010, Hryhorczuk et al., 2013, Stunkard and Wadden, 1992), with the latter being one of the most common behavioral traits in obese patients (Gariepy et al., 2010). Increased anxiety-like behavior has been reported in rodents genetically predisposed to develop obesity, e.g., *db/db* mice (Dinel et al., 2011), and in high-fat diet (HFD)-induced obesity (Heyward et al., 2012, Mizunoya et al., 2013). Processes such as inflammation (Capuron and Miller, 2011, Lasselin and Capuron, 2014), altered hormone signaling (Ulrich-Lai and Ryan, 2014), and stem cell dysfunction (Anacker and Hen, 2017, Gao et al., 2017) have been speculated to underlie obesity-related anxiety, but the underlying mechanisms have not been identified. Here, we investigate the hypothesis that anxiety-like behavior in obesity can be caused by increased senescent cell burden.

Cellular senescence is an irreversible cell-cycle arrest caused by a range of stresses, including telomere dysfunction (d'Adda di Fagagna et al., 2003), oxidative stress (Passos et al., 2010), inflammation (Jurk et al., 2014), intracellular accumulation of damage (Ogrodnik et al., 2018), and metabolic dysfunction (Wiley and Campisi, 2016). Senescent cells display a variety of markers, including telomere-associated DNA damage foci (TAF) (Hewitt et al., 2012), increased activity of lysosomal senescence-associated β-galactosidase (Dimri et al., 1995), chromatin changes (senescence associated heterochromatin foci, SAHF) (Narita et al., 2003), and frequently increased expression of the cyclin-dependent kinase inhibitor proteins, p16^Ink4a^ and p21^Cip1^.

While cell senescence is a potent tumor suppression mechanism (Muñoz-Espín and Serrano, 2014), over the long term, accumulation of senescent cells may impede the regeneration and maintenance of renewable tissues and, therefore, contribute to tissue aging. Additionally, senescent cells secrete a number of inflammatory cytokines, chemokines, and matrix proteases (the senescence associated secretory phenotype, SASP) (Coppé et al., 2008). The SASP is thought to have evolved as a means for senescent cells to communicate with the immune system in order to orchestrate senescent cell clearance and stimulate progenitor cells to repair tissues (Tchkonia et al., 2013). However, chronic exposure to the SASP leads to damage to neighboring healthy cells, thereby contributing to tissue dysfunction during aging and in age-related diseases (Acosta et al., 2013, Nelson et al., 2012, Xu et al., 2018).

Accumulation of senescent cells has been observed during obesity (Minamino et al., 2009, Ogrodnik et al., 2017, Schafer et al., 2016), during aging (Baker et al., 2016, Jurk et al., 2012, Wang et al., 2009, Xu et al., 2015, Yousefzadeh et al., 2018), and at the sites of pathogenesis in multiple chronic and age-related diseases (Tchkonia et al., 2013). Clearance of senescent cells can delay, prevent, or alleviate multiple age-related disorders (Kirkland and Tchkonia, 2017, Xu et al., 2018). These include age-related cardiac and vascular dysfunction (Childs et al., 2016, Roos et al., 2016), frailty (Baar et al., 2017, Baker et al., 2016, Xu et al., 2018, Zhu et al., 2015), hepatic steatosis (Ogrodnik et al., 2017), liver fibrosis (Moncsek et al., 2018), osteoporosis (Farr et al., 2017), osteoarthritis (Jeon et al., 2017), and pulmonary fibrosis (Schafer et al., 2016), among others. In the context of the brain, recent reports have shown that removing senescent cells improves phenotypes in mouse models of Parkinson’s disease (Chinta et al., 2018) and tau-dependent neurodegenerative diseases (Musi et al., 2018, Bussian et al., 2018). However, the relationship between senescence and neuropsychiatric disorders such as anxiety has not been investigated thus far.

Here, we demonstrate that in obesity, glial cells show increased markers of cellular senescence in the periventricular region of the lateral ventricle (LV), a region in close proximity to the neurogenic niche. Senescent glial cells in obese mice show excessive fat accumulation, a phenotype we termed “accumulation of lipids in senescence” (ALISE). Importantly, we show that clearance of senescent cells alleviates the obesity-related impairment in adult neurogenesis and decreases obesity-induced anxiety-like behavior. Our work suggests that targeting senescent cells may represent a new therapeutic avenue for treating obesity-induced neuropsychiatric dysfunction.

## Results

### Obese Mice Show Increased Anxiety-like Behavior Not Related to Body Mass

**Figure 1:**
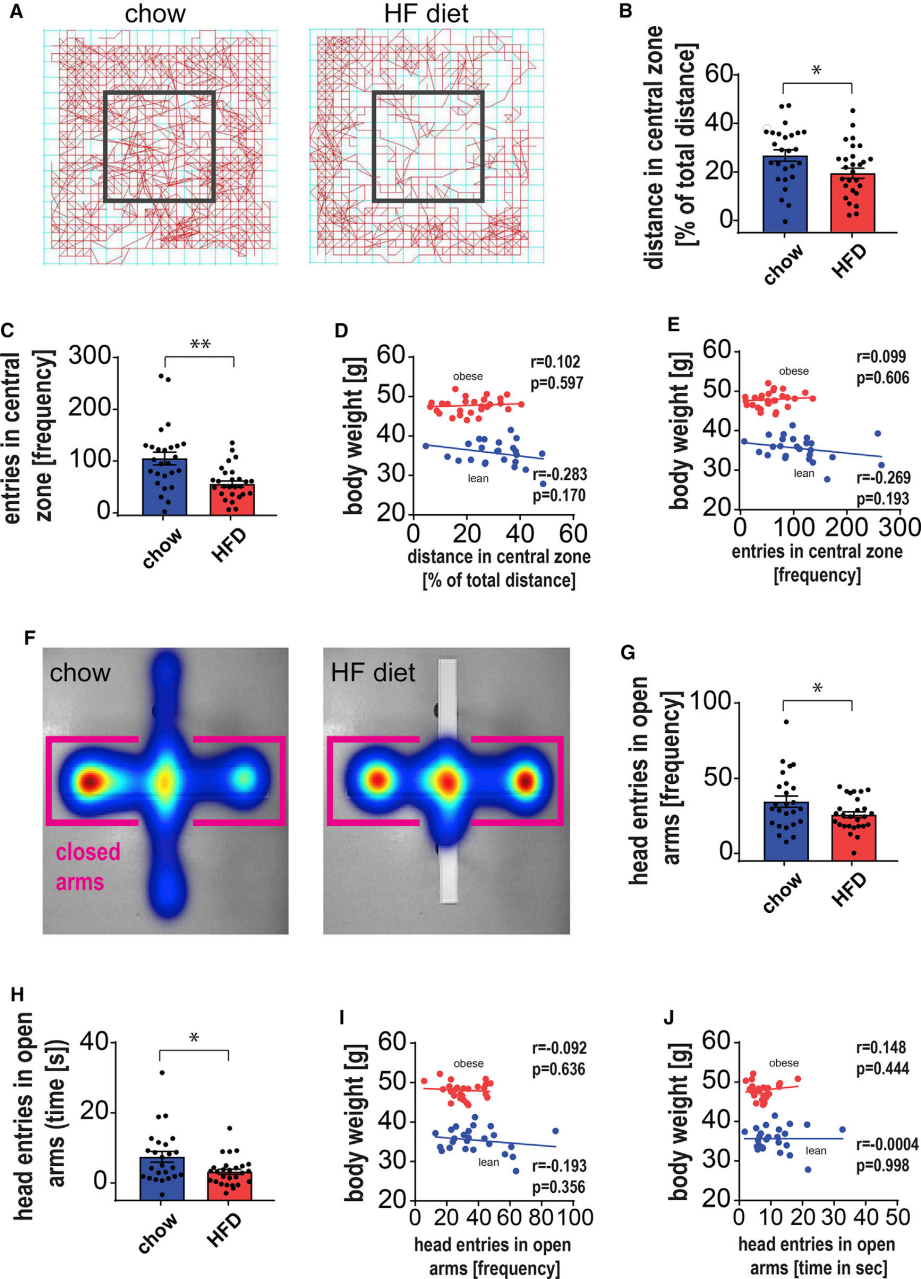
Obese Mice Exhibit Anxiety-like Behavior that Is Not Directly Related to an Increase in Body Mass Behavior changes were tested in the open-field (OF) chamber. Dark rectangle marks the central area (25% of total area). (A) Representative movement traces (red lines) for chow- and high-fat diet (HFD)-fed mice at 10 months of age (baseline). (B and C) Parameters recorded and analyzed in OF: (B) distance traveled in the central area (as a function of total distance traveled) (t_(54)_ = 2.359; p = 0.022) and (C) entries into the central area (U = 187, p = 0.0006). (D and E) No significant correlations (linear regression) were found in either chow or HFD animals between (D) body mass and the normalized distance mice traveled in the central area (r = 0.1024, p = 0.5972; r = −0.283, p = 0.1704) and (E) the number of entries into the central area (r = 0.09993, p = 0.606; r = −0.2692, p = 0.1933). (F) Representative heatmaps of time mice spent in the elevated plus maze (EPM) for chow and HFD mice. Closed arms of the maze are indicated by pink brackets. (G and H) (G) The frequency of head pokes into open arms (t_(36.66)_ = 2.045; p = 0.048) and (H) the time mice spent with their head in open arms (U = 230, p = 0.0209) are significantly decreased in HFD mice when compared to chow-fed mice. (I and J) No substantial correlations were found between body mass and EPM test parameters in chow and HFD mice: (I) frequency of head pokes into open arms (r = −0.09171, p = 0.6361; r = −0.1927, p = 0.356) and (J) time spent in open arms (r = 0.1477, p = 0.4444; r = −0.000444, p = 0.9983). Data are from n = 25–30 mice per group. Mean ± SEM plotted. ^∗^p ≤ 0.05 and ^∗∗^p ≤ 0.001.

In order to investigate the relationship between obesity and anxiety, 8-month-old C57Bl/6 mice were fed an HFD (60% of calories from fat) or standard chow diet for 2 months. As expected, we found that body weight and body fat content were increased in HFD mice in comparison to chow-fed controls (Figures S1A and S1B). To measure anxiety-like behavior, we first employed the open-field (OF) test. This test evaluates the tendency of mice to remain close to the walls and avoid open spaces (central zone), a phenomenon known as thigmotaxis, which is widely used as an indication of anxiety-like behavior (Simon et al., 1994). HFD-fed mice were less inclined to explore the central area of the OF test chamber than the peripheral zone (Figures 1A–1C and S1D), and likewise the total distance covered was significantly decreased in HFD animals during the test (Figure S1C). To account for decreased activity in obese animals, anxiety measurements were analyzed as a function of the total distance traveled during experimental testing (Figures 1B and 1D). As it has been shown that obesity impacts activity and exploratory behavior and contributes to anxiety-like behavior (Friend et al., 2017, Guillemot-Legris and Muccioli, 2017), we next investigated if body weight and body composition alone could explain the observed anxiety-like behavior. Linear regression analysis revealed no significant correlation between body weight or percentage of fat mass with anxiety-like behavior in HFD-fed mice (Figures 1D, 1E, and S1E–SIJ). This indicates that while weight gain is associated with the onset of anxiety-like behavior, once a certain weight is reached, no correlation between weight and anxiety is found, which suggests that factors apart from weight gain must play a role.

As an additional measurement of anxiety-like behavior, we used the elevated plus maze (EPM) test. The EPM is based on the animals’ natural fear of heights and open spaces. Increased anxiety-like behavior in the EPM test is manifested as a decrease in the number of head-pokes and entries into the open arms. We found that animals on the HFD had decreased entries into the open arms of the EPM (frequency and time) compared to lean animals (Figures 1F–1H), which is indicative of increased anxiety-like behavior. Similar to the OF test, there were no significant correlations between body and fat mass and anxiety parameters in either lean or HFD-fed animals (Figures 1I, 1J, S1K, and S1L).

### Pharmacogenetic and Pharmacologic Clearance of Senescent Cells Alleviates Obesity-Related Behavioral Changes

Several recent reports have demonstrated that the removal of senescent cells can alleviate a large array of age-related disorders (Kirkland and Tchkonia, 2017). Obesity has been associated with the accumulation of senescent cells in fat tissue (Minamino et al., 2009, Schafer et al., 2016, Tchkonia et al., 2010) and the liver (Ogrodnik et al., 2017). However, a link between obesity-induced senescence and anxiety has not been investigated. Therefore, we investigated whether senescent cells may contribute to anxiety-like behavior during obesity by using the INK-ATTAC mouse model. This model allows the selective suicide gene-mediated ablation of highly p16^Ink4a^-expressing cells, many of which are senescent, upon administration of the drug AP20187 (AP), which cross-links the ATTAC fusion protein, thereby activating its caspase 8 moiety to induce apoptosis (Baker et al., 2011, Xu et al., 2015).

**Figure 2:**
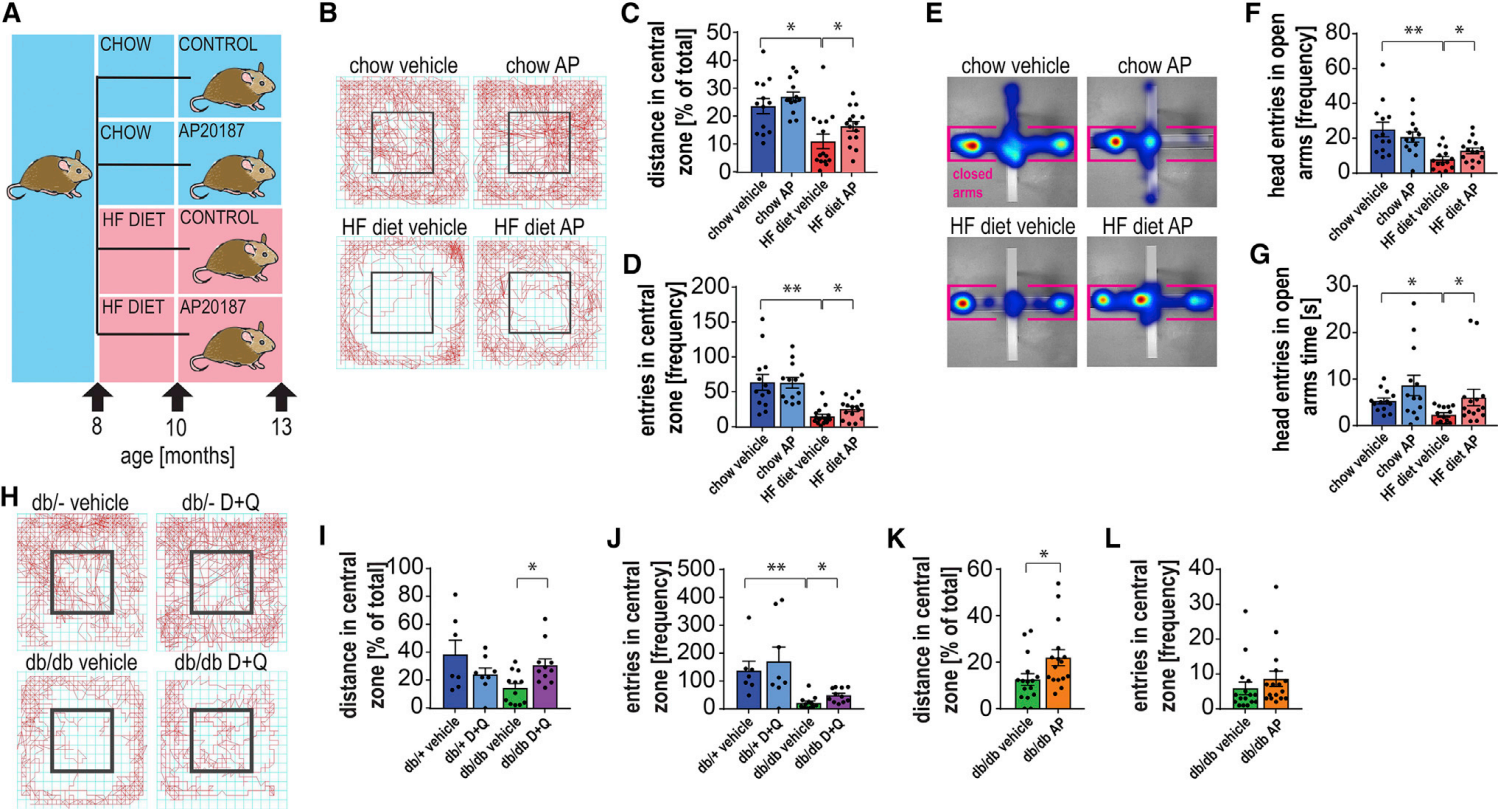
Pharmacogenetic and Pharmacologic Clearance of Senescent Cells from Obese Mice Alleviates Obesity-Related Behavioral Changes (A) Eight-month-old C57Bl/6^(INK-ATTAC)^ male mice were split into four groups and assigned to chow (n = 24) or high fat (HF, n = 30) diets and treated at 10 months of age with vehicle (n = 12 for chow and n = 15 for HF) or AP20187 (n = 12 for chow and n = 15 for HF) until 13 months of age. (B–D) (B) Open-field (OF) testing: representative movement traces (red lines) of lean and obese INK-ATTAC mice treated with/without AP20187 (AP) indicates that anxiety-like behavior of HFD mice can be alleviated by AP treatment as measured by (C) the normalized distance traveled in the central area of the OF box (U = 36, p = 0.0037; U = 64, p = 0.0453) and (D) increased frequency of entries into the central area. Alleviation of anxiety-like behavior of HFD mice was also observed in the EPM test (U = 10.5, p < 0.0001; U = 64.5, p = 0.0463). (E) Representative heatmap images of the time mice spent in open and closed sections of the EPM. Parameters were registered by EthoVision software. (F and G) (F) Frequency of head pokes into the open arms (U = 17.5, p < 0.0001; t_(27)_ = 2.1; p = 0.0452) and the (G) time mice spent with their heads in the open area were significantly reduced with HFD and increased with AP (U = 26, p = 0.001; U = 59.5, p = 0.0472). (H) Representative movement traces (red lines) of *db/db* and heterozygous control mice with or without treatment with senolytic cocktail, dasatinib + quercetin (D+Q) in the OF test during a 30-min trial. (I and J) Data indicate an improvement in the anxiety-like behavior of *db/db* mice upon D+Q treatment determined by OF test parameters: (I) normalized distance traveled in the middle area (t_(7.259)_ = 2.269, p = 0.0562; t_(21)_ = 3.001, p = 0.0068) and (J) frequency of entries into the middle area of the OF box (U = 1, p < 0.0001; U = 24.5, p = 0.009). (K and L) In the OF test, INK-ATTAC;*db/db* mice (K) displayed a significant difference in the distance traveled in the central area after treatment with AP20187 compared to vehicle (U = 61; p = 0.0106), but (L) the number of entries into the central area was not significantly increased after treatment with AP (U = 85.5; p = 0.1096). Data are from n = 12–15 mice per group for (A)–(G); n = 8–12 mice per group for (H)–(J); n = 5–9 mice per group for (K)–(L). Mean ± SEM plotted. ^∗^p ≤ 0.05 and ^∗∗^p ≤ 0.001.

We repeatedly treated chow- and HFD-fed 10-month-old mice with AP or vehicle (Figure 2A) over the duration of 10 weeks, which resulted in no significant changes in body weight (Figure S2A) or body composition (data not shown). To measure anxiety-like behavior, we first used the OF test and confirmed our previous observation that animals on the HFD were less inclined to explore the center of the open field chamber than the periphery, as measured by the distance traveled (Figure 2C) and entries (Figure 2D) into the central zone. Furthermore, HFD-fed mice traveled significantly less throughout the duration of the tests, covering a smaller total distance (Figure S2C). To take this into account, most measured parameters (as indicated) were expressed as a function of the total distance traveled. Clearance of p16^Ink4a^-positive cells with AP reduced HFD-induced anxiety-like behavior as measured by distance covered in the central zone (Figures 2B and S2B) and entries into the central zone (Figure 2C). However, AP treatment did not affect the total distance traveled (Figure S2C) or any of the aforementioned parameters in the chow-fed mice (Figures 2B–2D and S2C).

Next, we assessed anxiety-like behavior with the EPM. As previously noted, obese animals avoided entries into the open arms of the EPM (showing lower frequency of entries and less time spent) compared to lean animals (Figures 2E–2G, S2D, and S2E), indicating increased anxiety-like behavior. Consistent with the data from the OF test, AP treatment significantly decreased the anxiety-like phenotype in obese animals as indicated by the frequency of head entries into the open arms of the EPM (Figures 2E–2G, S2D, and S2E). Other cognitive functions, such as short-term memory (Figures S2F and S2G) measured by the Stone’s maze test, were not altered by the HFD or AP treatment. Altogether, these results show that specific elimination of p16^Ink4a+^-senescent cells from obese INK-ATTAC mice alleviates anxiety-like behavior but has no effect on memory performance.

To exclude off-target effects of the drug AP, wild-type C57Bl/6 mice were treated with the drug and tested for anxiety-like behavior. Consistent with previous studies, wild-type mice showed a significant difference between chow and HFD in the OF test before the start of the treatment (Figure S2H), but no difference was observed in the HFD-fed mice after treatment with AP (Figure S2I).

In addition to the HFD-fed mice, we conducted complementary experiments in *db/db* mice in which obesity is caused by a point mutation in the leptin receptor gene *lepr*, leading to spontaneous type 2 diabetes (Wang et al., 2014). We treated these mice intermittently for 2 months with the senolytic drug cocktail, Dasatinib and Quercetin (D+Q) (Zhu et al., 2015). *db/db* mice have significantly increased body weights and adipose depot weights when compared to lean *db*^+/−^ heterozygous littermates, but interestingly, body weight did not change over the course of D+Q treatment (Figures S2J and S2K).

Similar to HFD-fed mice, *db/db* mice exhibited increased anxiety-like behavior as assessed by the OF test (Figures 2H–2J). We observed that the total distance covered (Figure 2I) and the number of entries (Figure 2J) into the central zone were reduced in *db/db* mice compared to their non-obese, *db*^+/−^ heterozygous littermates, a phenotype which could be significantly alleviated by treatment with D+Q. While obese *db/db* mice covered a shorter total distance in comparison to their lean littermates, D+Q treatment did not change the total distance covered in the *db/db* or *db/db*^*+/−*^ mice (Figures S2K and S2L).

Finally, we confirmed these results in a cohort of double-transgenic, INK-ATTAC;*db/db* mice. Similarly to the treatment with D+Q, genetic clearance of p16^Ink4a^-positive senescent cells in INK-ATTAC;*db/db* mice did not alter body weight, body composition, or activity (Figures S2M–S2O). Importantly, clearance of p16^Ink4a^-positive senescent cells in INK-ATTAC;*db/db* mice reduced anxiety-like behavior as assayed by the OF test (Figures 2K and 2L). To exclude off-target effects of the drug AP, *db/db* mice were treated with AP and no off-target effects on anxiety-like behavior were observed in OF testing (Figure S2P). Similar to our previous observations, linear regression analysis showed no significant correlations between body weight and anxiety-like behavior in HFD-fed INK-ATTAC (with and without AP) or *db/db* (with or without AP or D+Q) mice (Figures S2Q–S2S).

In conclusion, our data show that pharmacological or pharmacogenetic clearance of senescent cells in two different models of obesity significantly alleviates anxiety-like behavior.

### Pharmacological and Pharmacogenetic Senolytic Approaches Reduce Senescent Cell Burden and Alleviate Systemic Inflammation

To investigate the effectiveness of senescent cell clearance in HFD mice, we measured senescence markers in the perigonadal adipose tissue, a tissue previously shown to exhibit a marked increase in the number of senescent cells with age (Schafer et al., 2016, Tchkonia et al., 2010, Xu et al., 2015).

**Figure 3:**
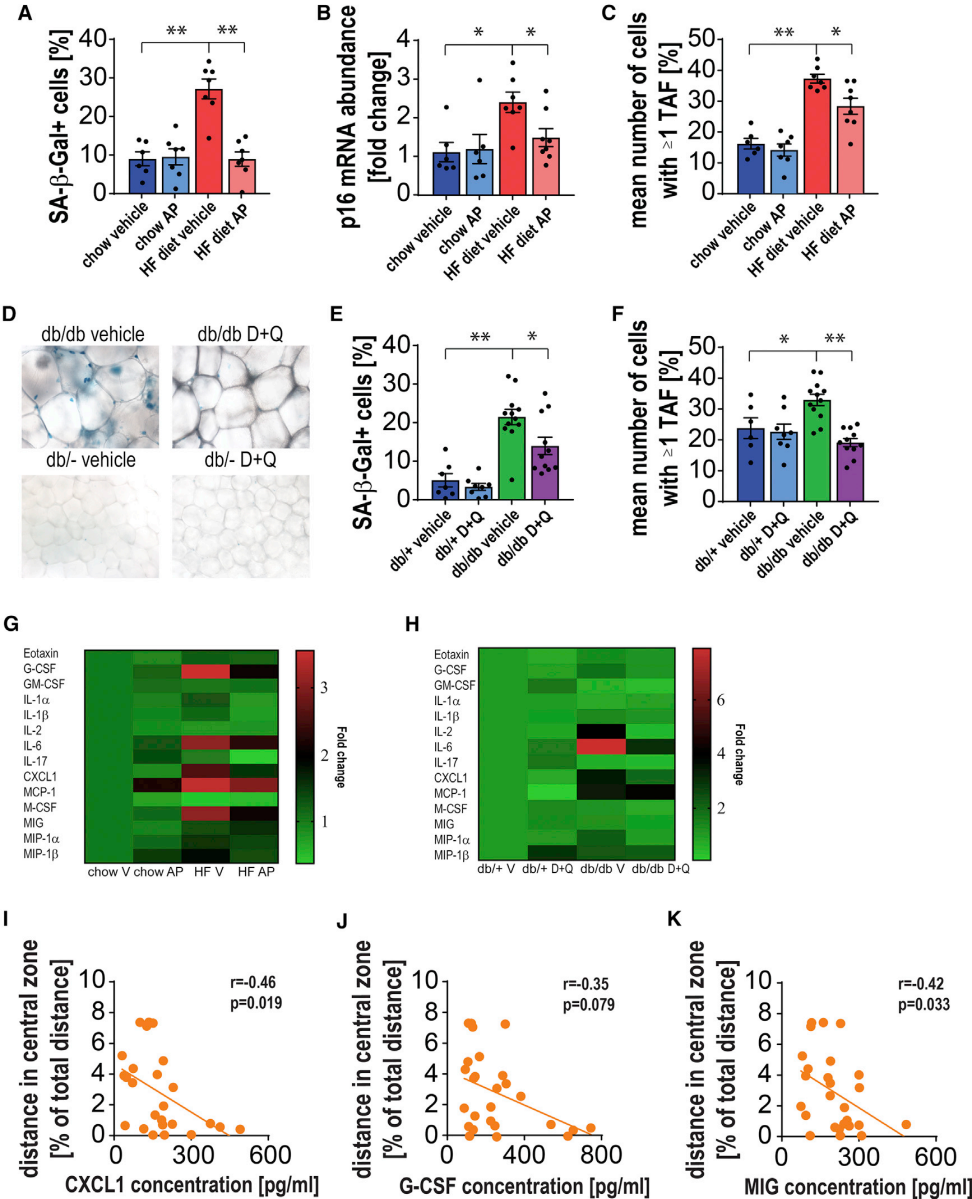
Clearance of Senescent Cells from Obese Animals Reduces Circulating Cytokine Levels (A) Quantification of percentage of senescence-associated beta-galactosidase (SA-β-Gal)-positive cells in perigonadal adipose tissue shows increased values in HFD-fed animals and complete rescue after treatment with AP20187 (t_(11)_ = 5.563, p = 0.0002; t_(12)_ = 5.739, p < 0.0001). (B and C) Senescence markers (B) p16 (measured by RT-PCR) (U = 5, p = 0.0221; t_(13)_ = 2.631, p = 0.0208) and (C) telomere associated DNA damage foci (TAF) (t_(11)_ = 9.495, p < 0.0001; t_(13)_ = 2.913, p = 0.0121) show a similar pattern. (D and E) (D) Representative images and (E) quantification of SA-β-Gal activity in perigonadal adipose tissue of *db/db* and *db/+* mice shows that the percentage of SA-β-Gal-positive cells increases in *db/db* mice compared to *db/+* and is significantly reduced after treatment with the senolytic cocktail, D+Q (t_(17)_ = 5.615, p < 0.0001; t_(21)_ = 2.504, p = 0.0206). (F) Frequencies of TAF-positive cells in perigonadal adipose tissue of *db/db* mice increased significantly in comparison to *db/*+ mice and decreased after D+Q treatment (t_(16)_ = 2.612, p = 0.0189; t_(21)_ = 5.937, p < 0.0001). (G) Cytokine protein expression [fold change] in blood plasma from HFD animals treated with/without AP20187. (H) Cytokine expression [fold change] in blood plasma from *db/db* animals treated with/without AP20187. (I–K) Linear regression analysis between anxiety markers and cytokines in blood plasma showed a significant negative correlation between (I) Cxcl-1 (r = −0.4663, p = 0.0188), (J) G-Csf (r = −0.3507, p = 0.079), (K) Mig (r = −0.4205, p = 0.0325), and the distance traveled in the central zone of the OF box in HFD-fed mice. Data are from n = 6–9 mice per group for (A)–(C) and (G); n = 7–12 mice per group for (E), (F), and (H); n = 27 mice per group for (I)–(K). Mean ± SEM plotted. ^∗^p ≤ 0.05 and ^∗∗^p ≤ 0.001.

We found that the senescence markers SA-β-Gal, p16, and TAF were increased in INK-ATTAC mice on HFD and were significantly reduced upon administration of AP (Figures 3A–3C). p21 and γ-H2A.X were increased in HFD animals but were not significantly changed after AP treatment (Figures S3A and S3B). Similarly, we found that *db/db* mice had an increased burden of senescent cells in perigonadal fat (measured by SA-β-Gal and TAF frequency) compared to *db/db*^*+/−*^ mice. This was significantly reduced by treatment with the senolytic cocktail, D+Q (Figures 3D–3F).

Given that the senolytic approaches we applied act systemically, it is possible that they reduce pro-inflammatory SASP factors that can penetrate the blood-brain barrier and therefore impact the brain. To investigate this possibility, we analyzed blood plasma for a large array of SASP factors. Evaluation of circulating cytokines in the bloodstream of chow- and HFD-fed INK-ATTAC mice revealed that HFD resulted in the up-regulation of known SASP factors such as G-Csf, Il-1α and Il-1β, Kc/Cxcl1, Mcp-1, Mig, and Tnf-α, which were then downregulated upon AP treatment (Figure 3G). Similarly, SASP factors in the blood plasma of *db/db* mice were increased in comparison to lean *db/db*^*+/−*^ littermates and reduced after treatment with D+Q (Figure 3H). As peripherally derived cytokines have been shown to inhibit neurogenesis and to drive anxiety and depression (Anacker and Hen, 2017, Gao et al., 2017, Li et al., 2014), we correlated the expression of cytokines in the bloodstream with parameters of anxiety-like behavior measured by OF testing. We observed significant negative correlations between the plasma levels of Cxcl1, G-Csf, and Mig and different anxiety-like measurements in AP-treated HFD mice (Figures 3I–3K and S3C) and D+Q-treated *db/db* mice (Figures S3D and S3E), whereas no correlation was found for Tnf-α, Il-6, and Mcp-1 (Figures S3F–S3H). These results led us to further investigate the impact of systemic factors on the observed anxiety phenotype. As Cxcl1 is the cytokine that best correlates with anxiety-like behavior and has been previously associated with obesity (Nunemaker et al., 2014), we injected lean animals with recombinant murine Cxcl1. As expected, injection of Cxcl1 led to increased levels of circulating Cxcl1 (Figure S3I). Additionally, increased plasma Cxcl1 resulted in a slight decrease in body weight but did not alter body composition (Figures S3J and S3K). Examination of anxiety-like behavior using the OF (Figure S3L) and EPM (Figure S3M) tests showed no difference between treated and non-treated animals. To further examine the role of Cxcl1 in anxiety-like behavior, we treated HFD mice with reparixin, which inhibits the Cxcl1 receptors Cxcr1 and Cxcr2. Mice on reparixin had a small increase in body weight (Figure S3N), but no difference in body fat (Figure S3O), in comparison to non-treated mice. Again, we observed no difference in behavior between the treated and non-treated groups when animals were tested in the OF box (Figure S3P) or the EPM (Figure S3Q). These results suggest that Cxcl1 alone is not sufficient to induce an anxiety-like phenotype; however, they do not exclude the possibility that other soluble SASP factors are involved in the process.

Recently, it has been shown that transplantation of relatively low numbers of senescent cells in young animals resulted in physical dysfunction measured by Rotarod performance, grip strength, or endurance compared to the transplantation of non-senescent cells (Xu et al., 2018). Importantly, this study showed that the transplantation of senescent cells resulted in long-lasting systemic effects in tissues located distantly from where senescent cells were injected.

To test the hypothesis that senescent cells can induce anxiety-like behavior via systemic effects, we transplanted senescent or non-senescent cells into lean mice and assessed behavior and physical function 6 and 12 weeks later (Figure S3R). We confirmed our previous observations that transplanted senescent cells reduced physical function (Xu et al., 2018), as measured by Rotarod (Figure S3S), but had no effect on anxiety-like behavior using the OF test (Figures S3T–S3W). Together, these experiments suggest that presence of senescent cells elsewhere in the body is not sufficient to induce an anxiety-like phenotype in mice.

### Senolytic Treatment Reduces the Frequency of Senescent Cells in the Amygdala and Hypothalamus, but Not in Other Regions of the Brain

Our previous results led us to hypothesize that obesity could induce senescence specifically in the brain, thereby contributing to an anxiety-like phenotype. Given the reported occurrence of senescence markers in neurons and glial cells of aging mice in the cortex, cerebellum, and hippocampus (Jurk et al., 2012, Kang et al., 2015), we first assessed markers of senescence in these regions of the brains of obese and lean INK-ATTAC mice treated with and without AP. We found no differences in the senescent markers p21, p16, γH2A.X, or TAF among any of the experimental groups (Figures S4A–S4D). This is consistent with the absence of differences in memory and learning among any of the experimental groups as assessed by Stone’s maze (Figures S2F and S2G).

**Figure 4:**
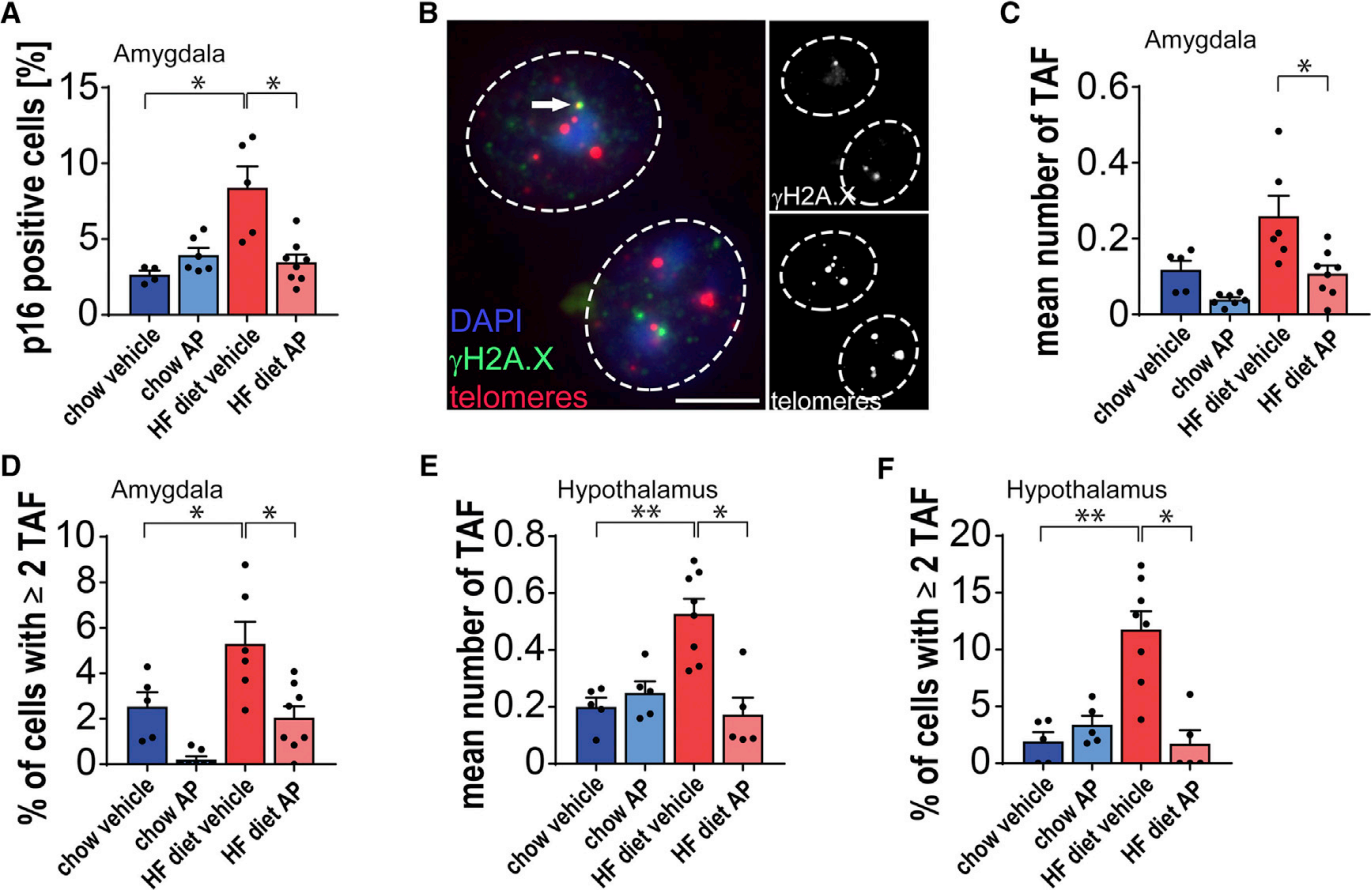
Markers of Senescence in the Amygdala Are Reduced after Treatment with AP20187 (A) p16-positive cells were measured by RNA-ISH in the basomedial layer of the amygdala (t_(9)_ = 2.627, p = 0.0275; t_(12)_ = 2.918, p = 0.0129). (B) Representative images showing telomere-associated DNA damage foci (TAF), (blue = DAPI, red = telomeres, green = γ-H2A.X, white arrow indicates TAF; scale bars, 5 μm) in 3-μm-thick paraffin embedded brain sections. (C and D) (C) Mean number of TAF (t_(9)_ = 2.221, p = 0.0535; t_(12)_ = 2.874, p = 0.014) and (D) percentage of NeuN^pos^ cells with 2 or more TAF (t_(4.298)_ = 3.943, p = 0.0147; t_(11)_ = 3.854, p = 0.0027) was increased in HFD INK-ATTAC mice and was significantly reduced after AP20187 treatment in the basomedial layer of the amygdala. (E and F) (E) Mean number of TAF (t_(11)_ = 4.449, p = 0.001; U = 2, p=0.0062) and (F) percentage of NeuN^neg^ cells with 2 or more TAF (t_(11)_ = 4.501, p = 0.0009; U = 1, p = 0.0031) were increased in HFD INK-ATTAC mice and significantly reduced after AP20187 treatment in the hypothalamus in close proximity to the 3^rd^ ventricle. Data are from n = 5–6 mice per group for (B)–(D); Mean ± SEM plotted. ^∗^p ≤ 0.05 and ^∗∗^p ≤ 0.001.

Interestingly, assessment of senescent cells in the amygdala, a region of brain associated with emotional responses such as anxiety and fear (Adhikari et al., 2015), revealed a significant increase in the number of p16^Ink4a^-positive cells in HFD-fed mice (Figure 4A). TAF-positive neurons in the basomedial layer of the amygdala were significantly increased in HFD mice and significantly decreased by treatment with AP (Figures 4B–4D). Next, we analyzed the burden of senescent cells in the hypothalamus close to the 3^rd^ ventricle and found that NeuN^neg^ (Figures 4E and 4F) and NeuN^pos^ (Figures S4E and S4F) senescent cells were significantly increased in HFD compared to lean mice and were significantly decreased by treatment of HFD-fed mice with AP.

Together, these data indicate that HFD does not induce senescence in regions of the brain implicated in learning, memory, or motor neuron control such as the cortex, cerebellum, and hippocampus. However, HFD induces senescence in the hypothalamus and amygdala, which may contribute to its effects on anxiety-like behavior, and treatment with AP reduced senescent cell abundance and attenuated these behavioral changes.

### Clearance of Senescent Cells Decreases Periventricular Accumulation of Lipid-Laden Glia in Obese Animals

Senescent cells have been shown to accumulate in obesity (Minamino et al., 2009, Ogrodnik et al., 2017, Schafer et al., 2016, Tchkonia et al., 2010), and their prevalence has been related to the accumulation of ectopic fat (Ogrodnik et al., 2017). Recently, it has been reported that cells accumulating lipid droplets in the brain occur in close proximity to the ventricles, and the number of these cells increases in mice with age (Shimabukuro et al., 2016), in Alzheimer’s disease (AD) patients, and in mouse models of AD (Hamilton et al., 2015). These data led us to investigate the connection between senescence and fat accumulation in the brain.

**Figure 5:**
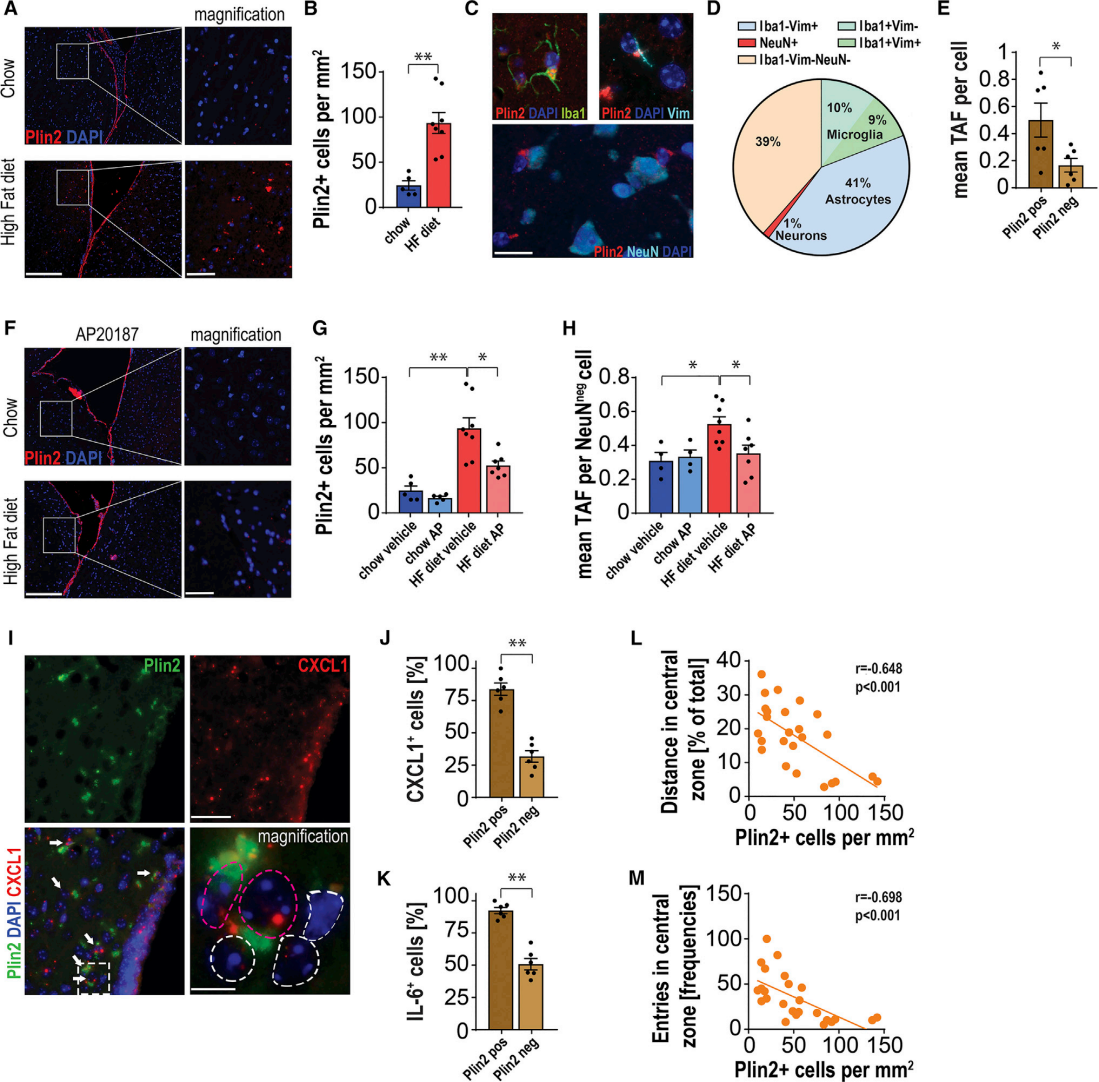
Obesity-Related Accumulation of Lipid Droplets in Senescent Periventricular Glia Is Reduced upon Senescent Cell Clearance (A) Representative images of perilipin 2 (Plin2) staining showing accumulation of cells exhibiting a buildup of lipid droplets in close proximity to the lateral ventricle (LV) of middle-aged, obese mice compared to their lean littermates. Scale bars, 200 μm/50 μm (for magn.) (B) Quantification of frequencies of Plin2^+^ cells in the proximity (up to 250 μm from the ependymal cell layer) to the LV in high-fat diet (HFD)-fed and lean mice (t_(11)_ = 4.48, p = 0.0009). (C) Representative images showing Plin2^+^ cells co-localizing with markers of microglia (Iba1; top left panel) and astrocytes (vimentin [Vim]; top right panel), but not with neuronal markers (NeuN; bottom panel, scale bars, 20 μm). (D) Pie chart shows cell-type composition of Plin2^+^ cells, determined after immunostaining for Plin2 and different cell-type markers, as shown in (C). (E) Periventricular Plin2^+^ glial cells show increased numbers of the senescence marker, telomere-associated foci (TAF). Quantification of the mean number of TAF per cell in non-neuronal (NeuN^neg^) Plin^+^ and Plin^−^ cells (t_(10)_ = 2.475, p = 0.0328). (F) Representative images show the LV of chow (top panel) and HFD AP20187-treated mice stained with Plin2, exhibiting reduced lipid droplets. Scale bars, 200 μm/50 μm (for magn.) (G) Quantification of cells containing lipid droplets (Plin2^+^) in the periventricular area of lean HF INK-ATTAC mice with or without AP20187 treatment (t_(11)_ = 4.48, p = 0.0009; t_(9.362)_ = 3.28, p = 0.0091). (H) Quantification of frequencies of NeuN^neg^, TAF-positive cells in the periventricular region of lean/HFD and vehicle- or AP20187-treated mice (t_(10)_ = 3.128, p = 0.0107; t_(13)_ = 2.693, p = 0.0184). (I) Representative images showing double staining for CXCL1 (RNA-ISH in red) and Plin2 (green) in the periventricular area of HF INK-ATTAC mice. White arrows indicate CXCL1 and Plin2 double positive cells (scale bars, 50 μm/10 μm for mag.). Cells magnified in panel 4 encircled in red show positive staining for Plin2 and CXCL1, whereas white-bordered cells are not double positive. (J and K) Quantification of cells positive for (J) Cxcl1 (t_(10)_ = 8.017, p < 0.0001) and (K) Il-6 (t_(10)_ = 8.298, p < 0.0001) stained by RNA-ISH indicate that Plin^+^ cells display significantly higher expression levels of the SASP factors, Il-6 and Cxcl1, than Plin^−^ cells. (L and M) Buildup of fat in periventricular brain region as assessed by Plin2 staining significantly correlates with parameters associated with anxiety-like behavior: (L) percentage of distance traveled in the central zone (percentage of central zone) (r = −0.6475, p = 0.0005) and (M) number of entries into the central zone (entries) in OF testing (r = −0.6976, p = 0.0001). Data are from n = 5–8 mice per group for (B), n = 6 mice per group for (E), (J), and (K), n = 4–8 mice per group for (G) and (H), and n = 25 mice per group for (L) and (M). Mean ± SEM plotted. ^∗^p ≤ 0.05 and ^∗∗^p ≤ 0.001.

Analysis of perilipin 2 (Plin2) expression (a protein that surrounds lipid droplets) in the brain of HFD-fed mice revealed a significant increase in Plin2^+^ cells (Figure S5A) located in close proximity to the LV compared to chow-fed controls (Figures 5A and 5B). Plin2^+^ cells were also detected around the 3^rd^ and 4^th^ ventricles and in the periaqueductal gray (PAG) matter (Figure S5B), but not in other brain regions (data not shown). Double-staining for Plin2 and the cell-type-specific markers, vimentin (Vim), Iba1, and NeuN, indicated that Plin2^+^ cells are mostly astrocytes (41%) and microglia (19%) (Figures 5C and 5D).

To investigate whether Plin2^+^ cells have features of senescence, we analyzed the senescence marker TAF in combination with immunostaining of Plin2. We found higher frequencies of TAF in Plin2^+^ cells (Figure 5E), while the total DNA damage did not change (Figure S5C). Together, our findings indicate that a HFD contributes to increased numbers of senescent cells in the periventricular region of the brain and that these cells preferentially accumulate fat. Given that we previously observed a similar phenomenon in senescent hepatocytes and fibroblasts (Ogrodnik et al., 2017), we termed this phenotype “accumulation of lipids in senescence” (ALISE).

To further investigate the impact of senescent cells on the buildup of fat in the brain, we used the INK-ATTAC mouse model (Baker et al., 2011). Treatment of HFD-fed INK-ATTAC mice with AP resulted in a significant reduction of Plin2^+^ cells (Figures 5F and 5G), as well as cells bearing senescence markers (Figure 5H). Furthermore, we analyzed the area occupied by lipid droplets in ependymal cells and the senescence marker TAF in glia in close proximity to the LV of *db/db* animals. We found that these were significantly increased in *db/db* in comparison to *db/db*^−/+^ animals and significantly reduced after treatment with the senolytic cocktail, D+Q (Figures S5D–S5F). Additionally, we assessed neuroinflammation by conducting RNA in situ hybridization against the SASP factors Cxcl1 and Il-6 in combination with immunostaining for Plin2 in close proximity to the LV of HFD mice (Figure 5I). Interestingly, we found that more than 80% of Plin2^+^ cells were also positive for Cxcl1 and Il-6 (Figures 5J and 5K).

Finally, anxiety markers in HFD animals, such as distance traveled in the central zone and entries into the central zone, were strongly negatively correlated with the abundance of Plin2^+^ cells in close proximity to the LV (Figures 5L and 5M). Together, these data support a causal link between the accumulation of lipid-laden senescent glial cells in obese animals and anxiety-like behavior.

### Suppression of the ALISE Phenotype Reduces Accumulation of Cytosolic Chromatin Fragments and the SASP

**Figure 6:**
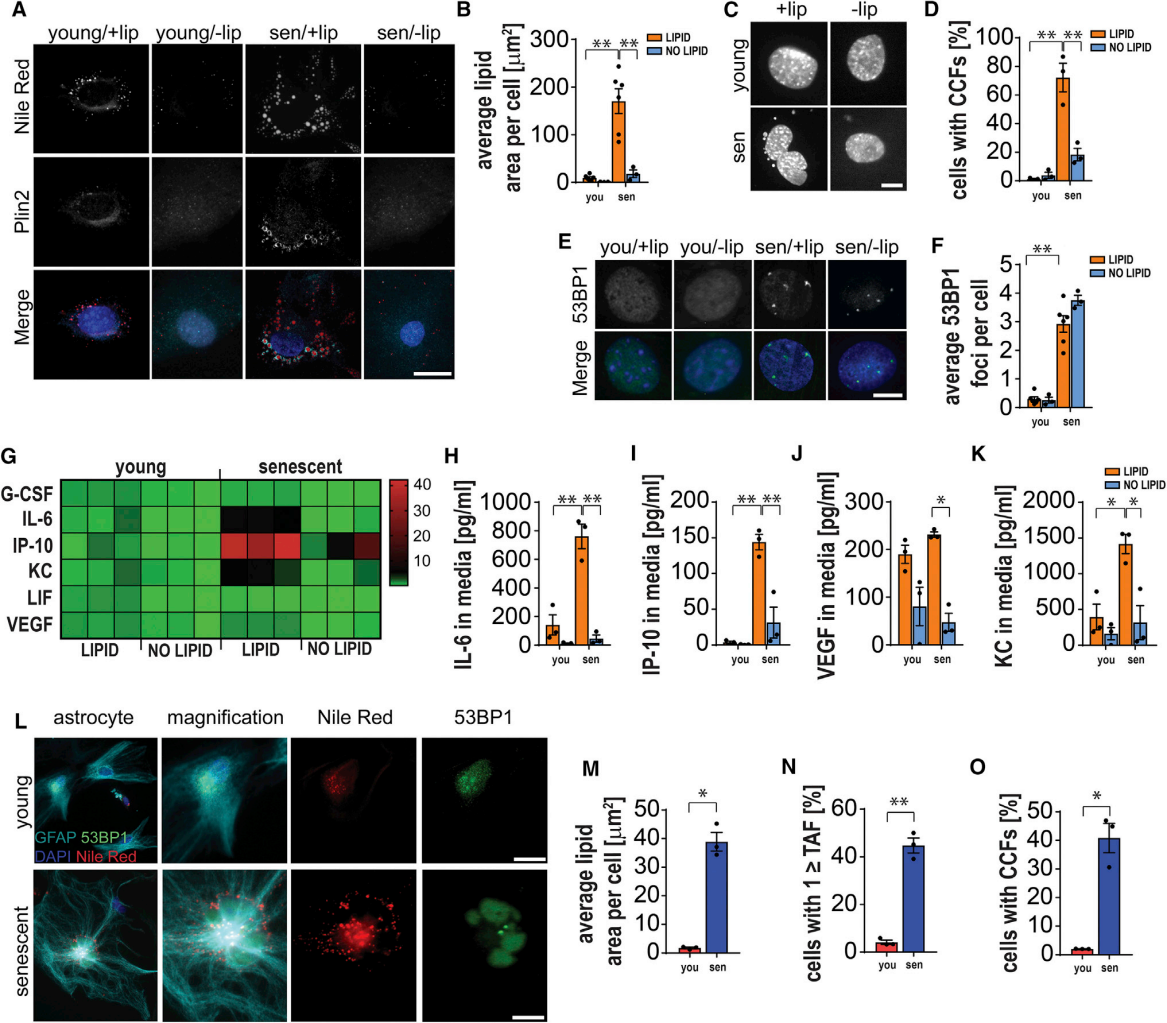
ALISE Phenotype Drives CCF Accumulation and the SASP Similar to glia in the brains of obese mice, mouse adult fibroblasts (MAFs) show an accumulation of lipids in senescence (ALISE) phenotype. (A–F) Depletion of lipids from culture media reduces area of lipid droplets in MAFs. (A) The ALISE phenotype in MAFs is characterized by an increased area of lipid droplets surrounded by Plin2 vesicles (scale bars, 20 μm). (B) Quantification of Nile Red-positive staining in non-senescent young (you) and senescent (sen) MAFs (p < 0.0001, p = 0.0004). (C and D) Suppression of the ALISE phenotype reduces frequencies of cytoplasmic chromatin fragments (CCF) in senescent fibroblasts (p < 0.0001, p = 0.0006) (scale bars, 10 μm), but (E) and (F) does not affect number of 53BP1 DNA damage foci (p = 0.0973) (scale bars, 10 μm). (G–K) Senescent fibroblasts show increased secretion of SASP components including Il-6, KC (Cxcl1), and Ip-10 (Cxcl10) that is alleviated upon suppression of the ALISE phenotype. (G) Heatmap shows fold change in secretion of SASP components in cell culture media over a 72-hr time period when compared to non-senescent fibroblasts cultured with lipid-containing media. Each square represents a separate biological replicate, i.e., MAFs isolated from a different mouse donor. Concentration of SASP components in culture media with and without lipids (ALISE phenotype suppression) for (H) Il-6 (p = 0.0003, p < 0.0001), (I) Ip-10 (Cxcl10) (p = 0.0002, p = 0.0008), (J) Vegf (p = 0.6407, p = 0.003), and (K) Kc (Cxcl1) (p = 0.0114, p = 0.0075). (L) Images show accumulation of lipid droplets in senescent but not non-senescent GFAP-positive astrocytes (scale bars, 20 μm). (M–O) Characterization of senescence in astrocytes. Senescent astrocytes show (M) the ALISE phenotype measured using Nile Red staining (t_(2.034)_ = 11.32, p = 0.0073), (N) increased frequencies of telomere dysfunction measured as the frequency of cells with 1 or more telomere associated DNA damage focus (TAF) (t_(4)_ = 12.45, p = 0.0002), and (O) increased frequencies of CCF (t_(2)_ = 7.551, p = 0.0171). Senescence was induced by X-ray irradiation (10Gy) and established within 14–21 days post-irradiation. Data are from n = 3–6 mice per group. Mean ± SEM plotted. ^∗^p ≤ 0.05 and ^∗∗^p ≤ 0.001.

To further investigate the impact of fat accumulation on cell senescence, we used mouse adult fibroblasts (MAFs) and induced senescence by X-ray irradiation as previously described (Jurk et al., 2014, Ogrodnik et al., 2017). We cultured senescent cells in the presence or absence of external sources of lipids (normal FBS versus lipid-deprived FBS). We found that in the absence of extracellular lipids, cells displaying the ALISE phenotype (assessed by lipophilic dye, Nile Red) were decreased (Figures 6A and 6B). Next, we investigated the impact of fat buildup on different markers of cellular senescence (Figures 6C–6K and S6A). Recently, it has been reported that senescent cells contain cytoplasmic chromatin fragments (CCFs) (Ivanov et al., 2013), which activate the DNA-sensing cGAS-STING pathway, a major driver of the SASP (Dou et al., 2017). Interestingly, we found that abrogation of the ALISE phenotype significantly reduced CCF in senescent cells (Figures 6C and 6D), but not the average number of DNA damage foci (Figures 6E and 6F) or bi-nuclearity (Figure S6A). Consistent with the hypothesis that enhanced lipid deposition impacts CCF and the SASP, we found that depriving cells of lipids resulted in a drastic reduction of several key components of the SASP, such as Il-6, Kc (Cxcl-1), Ip-10 (Cxcl-10), and Lix (Cxcl-5) (Figures 6G–6K). To investigate if these findings were restricted to MAFs, we conducted similar experiments in primary mouse astrocytes. Similar to MAFs, we found an increased buildup of fat, increased TAF, and higher numbers of CCF in senescent astrocytes (Figures 6L–6O).

Based on these data, we propose that excessive ALISE may be a contributor of genomic instability, resulting in the release of chromatin fragments and activation of the SASP.

### Impaired Neurogenesis in HFD Animals Is Rescued by Clearance of Senescent Cells

Ectopic buildup of lipid droplets in AD brains has been shown to induce dysfunction of neural stem cells residing within the subventricular zone (SVZ), suppress adult neurogenesis, and cause cognitive impairment (Hamilton et al., 2015).

In HFD mice, we observed that Plin2^+^ senescent glial cells are frequently found in close proximity to cells expressing doublecortin (Dcx), a marker of neuronal precursor cells and immature neurons (Figure S7A). Thus, we hypothesized that the presence of ALISE glial cells could impair adult neurogenesis in the SVZ.

**Figure 7:**
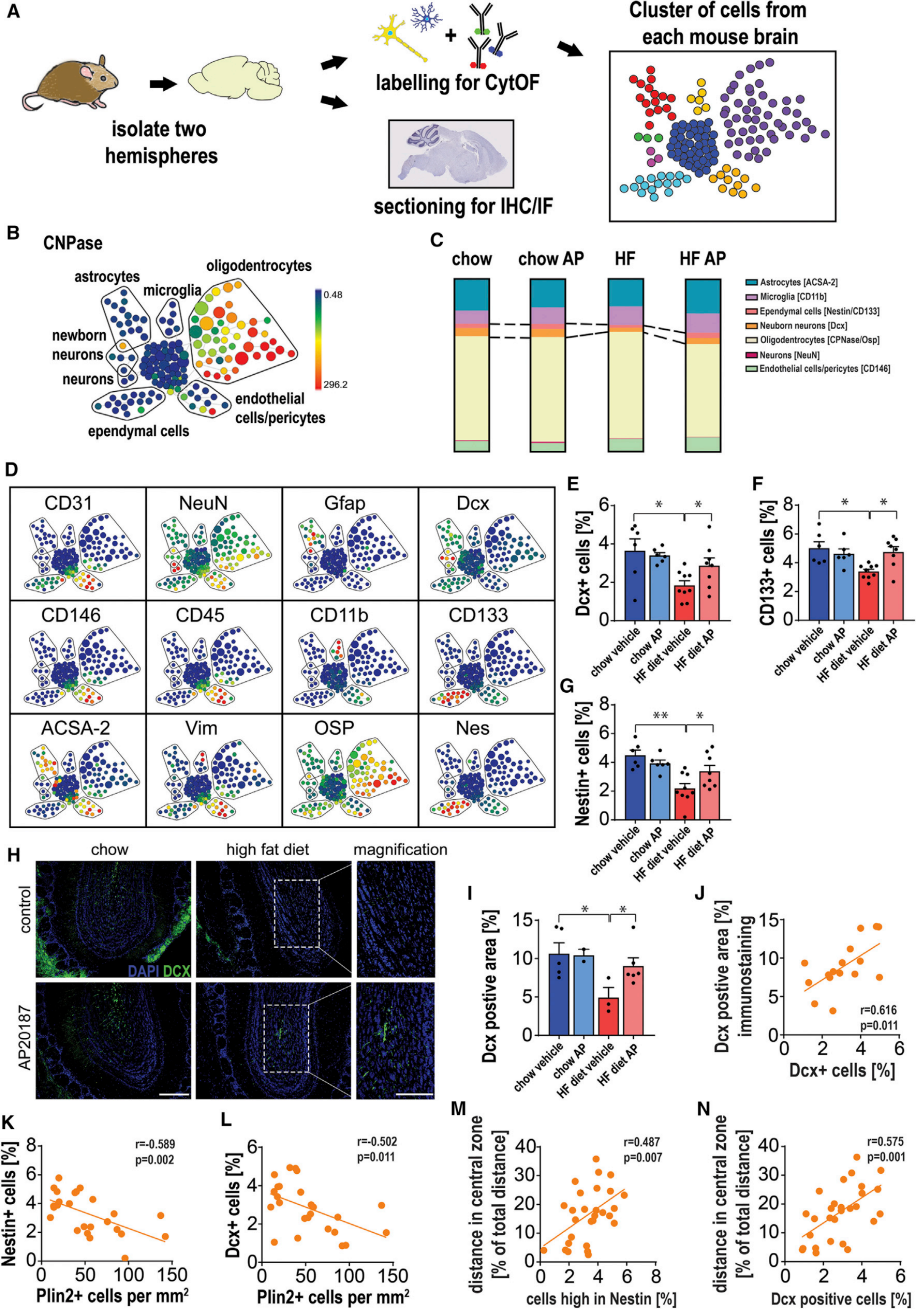
Clearance of Senescent Cells Partially Reverses the Neural Progenitor Cell Depletion Induced by Obesity (A) Each hemisphere of INK-ATTAC lean and high-fat (HF) mouse brains was either dissociated into a single-cell suspension or processed for IHC/IF. Dissociated brain cells were labeled with metal-conjugated antibodies and processed for cytometry by time of flight (CyTOF). (B and D) Spanning-tree progression analysis of density-normalized events (SPADE) was performed on brain cell populations identified by markers shown on the micrographs. Heatmap shows the intensity of the antibody signal, and the size of each spot is determined by the number of cells within this population. (C) CyTOF shows differences in brain cell populations of INK-ATTAC chow- and HFD-fed mice, which were treated with vehicle or AP20187. (E–G) Frequencies of cells expressing the markers (E) doublecortin (Dcx) (t_(13)_ = 3.057, p = 0.0092; t_(15)_ = 2.236, p = 0.041), (F) CD133 (t_(6.291)_ = 3.339, p = 0.0146; t_(9.674)_ = 3.383, p = 0.0073), and (G) Nestin (t_(13)_ = 4.405, p = 0.0007; t_(15)_ = 2.217, p = 0.0425) were quantified. (H) Representative images of Dcx staining in the olfactory bulb of chow- and HFD-fed mice treated with vehicle or AP20187 (scale bars, 250 μm). White boxes show magnified regions (scale bars, 200 μm). (I and J) (I) Quantitative analysis of the Dcx-positive area of the granular layer in the olfactory lobe of lean and obese INK-ATTAC mice (t_(6)_ = 2.707, p = 0.0352; U = 1, p = 0.0476) and (J) correlation between area occupied by Dcx^+^ cells in the granular layer of the olfactory bulb and frequencies of Dcx^+^ cells from the whole brain measured by CyTOF (r = 0.616, p = 0.0111). (K and L) Correlations (linear regression analysis) between frequencies of periventricular glia exhibiting accumulation of lipid droplets and frequencies of cells expressing markers of neurogenesis and ependymal cells (determined by CyTOF): (K) Nestin (r = −0.5887, p = 0.002) and (L) Dcx (r = −0.5022, p = 0.0105). (M and N) Correlations (linear regression analysis) between distance traveled in the central zone and the frequencies of cells expressing (M) Nestin (r = 0.487, p = 0.0074) and (N) Dcx (r = 0.5749, p = 0.0011) (determined by CyTOF). Data are from n = 5–9 mice per group for (C)–(G); n = 2–6 mice per group for (I); n = 11 mice per group for (J); n = 29 mice per group for (K)–(N). Mean ± SEM plotted. ^∗^p ≤ 0.05 and ^∗∗^p ≤ 0.001.

To test this hypothesis, we treated lean and obese INK-ATTAC mice with or without AP, as previously described. Following organ harvesting, we obtained single-cell suspensions from one brain hemisphere and analyzed them by cytometry by time of flight (CyTOF), which allows for mapping and discriminating between different brain cells including astrocytes, oligodendrocytes, microglia, neurons, ependymal cells, pericytes, and endothelial cells. We reserved the second brain hemisphere for histological analyses (Figures 7A and 7B).

We found that the brains of HFD-fed mice did not exhibit significant changes in the frequencies of oligodendrocytes (CNPase^+^ or OSP^+^), microglia (CD11b^+^, CD45^−^), mature neurons (NeuN^+^), or endothelial cells (CD31^+^ or CD146^+^) (Figures 7C and S7B–S7M). However, populations of neuronal precursor cells (Nestin^+^), immature neurons (Dcx^+^), and ependymal cells (CD133^+^) were significantly decreased in animals subjected to HFD feeding (Figures 7C–7G). Clearance of senescent cells by AP did not alter the frequencies of oligodendrocytes (CNPase^+^ or OSP^+^), microglia (CD11b^+^, CD45^−^), mature neurons (NeuN^+^), or endothelial cells (CD146^+^) (Figures 7C and S7B–S7M), but it significantly increased neuronal precursor cells, immature neurons, and ependymal cells (Figures 7C–7G). CD133^+^ ependymal cells have been reported to possess properties of neural stem cells and give rise to Dcx^+^ neuroblasts and interneurons in the olfactory bulb (Luo et al., 2015). AP treatment was sufficient to induce partial recovery of obesity-related stem cell depletion (Figures 7C and 7E) and to replenish CD133^+^ and Nestin^+^ cell abundance (Figures 7F and 7G). Analysis of cell populations in the brains of *db/db* mice showed a similar pattern as those of HFD-fed mice. Markers for immature neurons (Dcx^+^), ependymal cells (CD133^+^), and neuronal precursor cells (Nestin^+^) were all significantly up-regulated after senolytic treatment with D+Q (Figures S7N–S7P).

We validated these findings by performing immunostaining for Dcx (Figures 7H and 7I) in the olfactory bulb and EdU-pulse labeling (Figures S7S and S7T) in the SVZ of HFD animals. Analysis of Dcx^+^ cells in the olfactory bulb (performed in the same mice as in the CyTOF experiment) showed a positive correlation between the area occupied by Dcx^+^ cells in the granular cell layer of the olfactory bulb and the frequency of Dcx^+^ cells detected by CyTOF (Figure 7J). Quantification of Dcx^+^ cells (Figures S7U and S7V) and EdU^+^ cells (Figure S7W) in the sub-granular zone (SGZ) of the hippocampus did not show any effect of AP treatment (data not shown), implying that adult neurogenesis in the SVZ, but not in the SGZ, is affected by the presence of senescent cells. Interestingly, clearance of senescent cells led to a significant increase in the population of astrocytes (Figures S7G–S7I) in obese animals, whereas no differences between lean and obese animals were detected. This finding was further confirmed by immunostaining for Gfap (Figures S7Q and S7R) and may be related to increased brain plasticity supporting neurogenesis (Choi et al., 2016). Finally, we found that the frequencies of Plin2^+^ glia negatively correlated with the markers of ependymal cells and markers of adult neurogenesis, Nestin^+^ (Figure 7K) and Dcx^+^ (Figure 7L). Similarly, individual differences in the distance traveled in the central area were positively correlated with the frequencies of Dcx^+^ and Nestin^+^ cells (Figures 7M and 7N).

In summary, our data indicate that senescent cells play a causal role in the impaired neurogenesis induced by obesity. Targeting senescent cells in obese mice alleviates obesity-related anxiety-like behavior as a result of the clearance of periventricular fat accumulation and restoration of adult neurogenesis.

## Discussion

Obesity is a complex disorder and a major risk factor for many diseases and health problems, including heart disease, diabetes, stroke, high blood pressure, cancer, osteoarthritis, and cognitive impairment (Frasca et al., 2017). Additionally, decreased spatial memory, anxiety, and depression have been linked to obesity-associated brain inflammation (Guillemot-Legris and Muccioli, 2017). Previous studies have reported that HFD- and genetically induced obesity in mice results in anxiety-like behavior (Dinel et al., 2011, Heyward et al., 2012, Mizunoya et al., 2013). However, similar to other obesity-driven phenotypes such as physical inactivity (Friend et al., 2017), anxiety-like behavior is not simply caused by increased body mass. Interestingly, loss of body weight alone does not always correspond to improvements in mental health, e.g., after bariatric surgery (Canetti et al., 2016, Cunningham et al., 2012, Fisher et al., 2017, Kouidrat et al., 2017, Matini et al., 2014). Thus, the mechanistic basis of obesity-induced anxiety-like behavior is not yet fully understood.

HFD induces senescence in multiple organs, predominantly in perigonadal adipose tissue (Schafer et al., 2016, Tchkonia et al., 2010, Xu et al., 2015) but also in other organs including the liver (Ogrodnik et al., 2017). Until now, the role of HFD in the induction of senescence in the brain is largely understudied. Recently, several studies have uncovered a role for senescent glial cells in neurodegenerative diseases such as tau-dependent pathology and Parkinson’s disease (Bussian et al., 2018, Chinta et al., 2018, Musi et al., 2018).

Our data demonstrating a link between obesity, senescence, and anxiety-like behavior provide critical support for the potential feasibility of administering senolytics to treat obesity-associated anxiety-like behavior, provided that clinical trials validate this approach.

It should be mentioned that our experimental strategy does not distinguish which senescent cell types are responsible for inducing anxiety-like behavior. However, we found that senescent, pro-inflammatory glial cells are frequently found in close proximity to areas in the brain expressing markers of neuronal precursor cells and immature neurons, which suggests that they may exert negative effects on stem cells. While senescence can impact stem cells in a cell-autonomous fashion by affecting their proliferation and differentiation (Xu et al., 2015), it had been previously suggested that senescence can impact stem cells non-autonomously via the SASP (Krtolica et al., 2011, Pricola et al., 2009). Consistent with this hypothesis, we found that SASP factors in blood plasma and specifically in the brain were increased during obesity and significantly reduced following both the pharmacogenetic and pharmacological clearance of senescent cells. Nonetheless, neither injection of the major SASP factor Cxcl1 (which increased during obesity) nor inhibition of its receptors had an impact on anxiety-like behavior. Additionally, transplantation of senescent cells, which has been previously shown to induce systemic effects and result in long-lasting physical dysfunction in mice, did not have an impact on anxiety-like behavior. These data suggest that anxiety-like phenotypes driven by obesity may be a consequence of senescence occurring locally in specific regions of the brain. However, given the fact that there are as yet no means to eliminate senescent cells from specific regions of the brain, this hypothesis remains highly speculative.

Dysfunction of peripheral (Ambrosi et al., 2017, Cignarelli et al., 2012, Matsushita and Dzau, 2017) and neural stem cells (Li et al., 2014) has been linked to obesity and metabolic syndrome and inflammation and have been postulated to be among the factors connecting adult neural stem cell dysfunction to obesity (Li et al., 2014). Nevertheless, the underlying mechanisms remain unclear. We previously reported that reducing the abundance of senescent cells that accumulate with aging restores adipose (Xu et al., 2015) and bone stem cell function (Farr et al., 2017). Similarly, others have reported improvement in hematopoietic and hair follicle stem cell function (Chang et al., 2016, Yosef et al., 2016) and neurogenesis in a model of Parkinson’s disease (Chinta et al., 2018) following various senolytic treatments. Here, using CyTOF and EdU-pulse labeling, we demonstrate that clearing senescent cells in obese mice partially restores the neural stem cell pool.

A link between anxiety-like behavior in mice and neural stem cell function has been previously established (Drew and Huckleberry, 2017, Siopi et al., 2016). Our data suggest that the subventricular zone-olfactory bulb axis is involved in the therapeutic effects of senescent cell clearance. Functional inactivation of the main olfactory epithelium was shown to be sufficient to induce anxiety-like behavior in mice (Glinka et al., 2012). Additionally, adults with a history of childhood stress or major depressive disorders show reduced olfactory bulb volume (Croy et al., 2013, Negoias et al., 2010).

Although we show that expression of neurogenesis markers is negatively associated with anxiety-like behavior, we cannot exclude the possibility that it is anxiety itself that impairs neurogenesis. In a recent study, chronic corticosterone administration was shown to contribute to anxiety and depression and was sufficient to induce olfactory deficits and impair adult neurogenesis (Siopi et al., 2016).

In our study, we also made the novel observation that senescent glial cells accumulate lipids, a phenotype that also occurs in senescent fibroblasts and astrocytes cultured *in vitro*, which we termed ALISE. Hamilton et al. (2015) have shown that the accumulation of lipid droplets in the SVZ occurs in mouse models of AD and human AD patients. They also reported that these lipids are derived from the cerebrospinal fluid and that the presence of lipid droplets is associated with neuronal stem cell dysfunction in a mouse model of AD (Hamilton et al., 2015). Our data show that it is the presence of lipid-containing senescent cells, rather than accumulation of lipids per se, that contributes to impaired neurogenesis. Our data imply that it is the elimination of senescent cells, rather than the reduction of lipid availability, that improves neurogenesis and alleviates mouse anxiety-like behavior.

Mechanistically, lipid accumulation in senescent cells has been related to mitochondrial dysfunction and impaired fatty acid oxidation (Ogrodnik et al., 2017). Recent data have shown that mitochondrial dysfunction during senescence is a key effector of cellular senescence, playing a role in the stabilization of the growth arrest (Passos et al., 2010) and the induction of the SASP (Correia-Melo et al., 2016). Based on these findings and our data, we speculate that when lipids are available, senescent cells cannot effectively metabolize them, which in turn results in increased genomic instability, release of CCF, and induction of the SASP.

Despite increasing evidence that anxiety is associated with decreased quality of life, increased depression, and suicide in obesity (Wagner et al., 2013), there are currently no mechanism-based treatments available. Here, we show that cellular senescence provides a potential explanation for increased anxiety in obese individuals and that targeting senescent cells holds promise as a therapeutic strategy.

### Limitations of Study

While our study shows that senescent cells have an impact on anxiety, our experimental strategy was not capable of distinguishing which senescent cell types are responsible. Further studies using animal models where senescent cells can be cleared in a cell-type specific manner will be able to address this particular pitfall. Obesity does impact locomotion (obese animals move more slowly and tend to freeze more), which may induce some artifacts to the analysis of anxiety-like behavior. Future studies may use non-locomotion-based assays such as marble burying.

## STAR★Methods

### Key Resources Table

**Table:** 

REAGENT or RESOURCE	SOURCE	IDENTIFIER
**Antibodies**		

Lexa 555-azide	Life technologies	Cat: #A20012
Anti-Nestin mouse monoclonal antibody (for CyTOF)	Abcam	Cat # ab6142; RRID: AB_305313
Anti-CD133 rat monoclonal antibody (for CyTOF)	Biolegend	Cat # 141202; RRID: AB_10896423
Anti-Doublecortin mouse monoclonal antibody (for CyTOF)	Abcam	Cat # ab135349; Clone: 2G5
Anti-Perilipin 2 guinea pig polyclonal antibody	PROGEN	synthetic peptide (N-terminal aa 1-12 of human adipophilin/PLIN2)
Anti-53BP1 rabbit polyclonal antibody	Novus Biologicals	Cat # NB100-304; RRID: AB_10003037
Anti-GFAP guinea pig polyclonal antibody (for IHF)	Synaptic Systems	Cat # 173 004; RRID: AB_10641162
Anti-γ-H2A.X rabbit monoclonal antibody	Cell Signaling Technology	Cat # 9718; RRID: AB_2118009
Anti-Doublecortin rabbit antibody (for IHF)	Cell Signaling Technology	Cat # 4604; RRID: AB_561007
Anti-Glial Fibrillary Acidic Protein (GFAP) rabbit antibody (for IHC)	Original Manufacturer: Dako; Now: Agilent Technologies	Cat # N1506; RRID: AB_10013482
Anti- CD11b (Mac-1)	Fluidigm	Cat: #3154006B
Anti- CD45	Fluidigm	Cat: #3089005B
Anti- CD31	Fluidigm	Cat: #3165013B
Anti- CD146	Fluidigm	Cat: #3141016B
Anti-Vimentin	Abcam	Cat:# ab8978
Anti-ACSA-2	Miltenyi Biotec	Cat:# 130099138
Anti-GFAP	ebioscience	Cat:# 14-9892-82
Anti-NeuN	abcam	Cat:# ab177487
Anti-CNPase	abcam	Cat:# ab53041
Anti-OSP	abcam	Cat:# ab53041

**Chemicals, Peptides, and Recombinant Proteins**		

AP20187	MCE MedchemExpress.com	Cat: # HY-13992/CS-1953
EdU	Life technologies	Cat: #E10187
Dasatinib	LC Laboratories	Cat: # D-3307
Quercetin	Sigma	Cat: # Q4951
Recombinant CXCL1	Peprotech	Cat# 250-11
Reparixin L-lysine salt	MedChemExpress	Cat: # HY-15252

**Critical Commercial Assays**		

Adult Brain Dissociation Kit	Miltenyi Biotec, CA	Cat: #130-107-677
Mouse Cytokine Array / Chemokine Array 31-Plex	Eve Technologies; Canada	MD31
M-MLV Reverse Transcriptase kit	Life Technologies	Cat# 28025013
CyTOF for conjugating antibodies with metals	Fluidigm	Cat#:201300
RNA-ISH kit	Acdbio	Cat: #322350

**Deposited Data**		

CyTOF data	Mendeley.com	http://dx.doi.org/10.17632/w4gnsvjdf6.2

**Experimental Models: Cell Lines**		

Primary Mouse (ear) Adult Fibroblasts (MAFs)	Ogrodnik et al., 2017, Jurk et al., 2014	N/A
Primary Mouse Embryonic Astrocytes	see Method Details section	N/A

**Experimental Models: Organisms/Strains**		

Mouse: C57BL/6 INK-ATTAC	Baker et al. (2011)	N/A
Mouse: BKS.Cg-Dock7m Homozygous Leprdb/J (*db/db*) and BKS.Cg-Dock7m Heterozygous Leprdb/J (*db/+*)	Jackson Laboratories	Stock No: 000697
Mouse: C57BL/6 INK-ATTAC *db/db* and *db/+* mice	Mayo	N/A
Mouse: C57BL/6 (Wt) mice	Jackson Laboratories	Stock No: 000664
Mouse: C57BL/6 Luc+ mice	Jackson Laboratories	Stock No: 025854
Mouse: C57BL/6 (Wt) mice (transplantation)	Dr. Kirkland lab (Mayo)	N/A

**Oligonucleotides**		

Primers for p16 (CDKN2A)	Applied Biosystems	Cat# 4331182; Assay ID: Mm00494449_m1
Primers for p21 (CDKN1A)	Applied Biosystems	Cat# 4331182; Assay ID: Mm04205640_g1
Primers for TATA-binding protein (TBP)	Applied Biosystems	Cat# 4331182; Assay ID: Mm01277042_m1
Telomere-specific TelC-Cy3 peptide nucleic acid probe	Panagene	Cat# F1002
RNA-ISH probes	Adcbio	Cat: # 407721 (Cxcl1), Cat: # 315891 (IL-6), Cat: # 400281 (eGFP)

**Software and Algorithms**		

Graphpad Prism 5 and 7	GraphPad Software	https://www.graphpad.com/scientific-software/prism/
SPADE TREE ANALYSIS	Cytobank	https://www.cytobank.org/

**Other**		

Fetal Bovine Serum (FBS) South America, Lipid Depleted	Biowest	Cat# S181L-500
Nile red	Sigma-Aldrich	Cat# N3013
Rodent Diet With 60 kcal% Fat	Research Diets	Cat# D12492
Rodent Chow Diet	Lab Diet (Lab Supply, Fort Worth, TX, US)	Cat# 5053
TaqMan Fast Universal PCR Master Mix	Life Technologies	Cat# 4352042

### Contact for Reagent and Resource Sharing

Further information and requests for resources and reagents should be directed to and will be fulfilled by the Lead Contact, Diana Jurk (jurk.diana@mayo.edu).

### Experimental Model and Subject Details

#### Animals

Experimental procedures were approved by the Institutional Animal Care and Use Committee at Mayo Clinic (protocol A26415). INK-ATTAC^+/-^ transgenic mice were generated and genotyped as previously described (Baker et al., 2011) based on experimental strategies devised by J.L.K., T.T., J. van Deursen and D. Baker at Mayo Clinic. Briefly, INK-ATTAC mice were produced and phenotyped at Mayo Clinic. Controls for the INK-ATTAC experiments were INK-ATTAC-null C57BL/6 background mice raised in parallel. C57BL/6 db/db and *db*/+ mice were purchased from Jackson Laboratories.

Mice were housed 2-5 mice per cage, at 22 +/-0.5°C on a 12-12 hour day-night cycle and provided with food and water *ad libitum*. Cages and bedding (autoclaved Enrich-o’Cobs (The Andersons Incorporated)) were changed once per week. For high fat diet-induced obesity studies, mice were randomly assigned to chow- or HFD-fed groups. Mice were fed the high fat diet for 2-4 months before experiments started. High fat food was purchased from Research Diets (cat no #D12492). 60% of calories in this high fat diet are from fat. Standard mouse chow diet was obtained from Lab Diet (cat no #5053). In all the experiments littermates of the same sex were randomly assigned to experimental groups.

INK-ATTAC mice were injected intraperitoneally (i.p.) with AP20187 (10mg/kg) (MCE MedchemExpress; Cat: # HY-13992/CS-1953) or vehicle (4% ethanol, 10% PEG-400, and 2% Tween-20 in distilled water) for 3 days every 2 weeks for a total of 8-10 weeks.

Senolytic-treated *db/db* mice were gavaged with Dasatinib (D; 5mg/kg) (LC Laboratories; Cat: # D-3307) and Quercetin (Q; 50mg/kg) (Sigma; Cat: # Q4951) or vehicle (60% Phosal, 10% ethanol, and 30% PEG-400) for 5 days every 2 weeks for 8 weeks.

For off target effect measurements *db/db* and HDF-fed mice (HFD for 2 months prior treatment) were injected intraperitoneally (i.p.) with 10mg/kg AP21087 or vehicle for 3 days every 2 weeks for 8 weeks.

Recombinant CXCL1 (Peprotech, #250-11) or vehicle (PBS) was administered to lean C57BL/6 *via* i.p. injection (5μg/kg in PBS) daily for 7 days. Two hours after the last injection mice were tested in open field and elevated plus maze and dissected afterwards.

Reparixin L-lysine salt (MedChemExpress, #HY-15252) or L-Lysine hydrochloride (MedChemExpress, #HY-N0470) dissolved in H_2_O was administered to obese C57BL/6 mice (fed for 2 months with HFD) *via* subcutaneous injection (30 mg/kg) twice per day for 2 weeks. Two hours after the last injection, mice were tested in open field and elevated plus maze and dissected afterwards.

Tissues from mice sacrificed at the indicated time points were snap-frozen in liquid nitrogen for biochemical studies or fixed in 4% Paraformaldehyde (PFA) for 24 hours prior to processing and paraffin embedding. Paraffin-embedded tissues were cut at 3μm or 10μm intervals.

#### Cell Culture

##### Mouse Adult Fibroblasts (MAF)

MAF were extracted from 3-5 month old male C57BL/6 mice. Ear clippings were transported and stored (not longer than 1h) in serum-free DMEM on ice. Punches were washed 3 times with serum-free media, finely cut and incubated for 2–3 h at 37°C in DMEM containing 2 mg/ml collagenase A. A single-cell suspension was obtained by repeated pipetting and passing through a 24-G fine needle. Cells were centrifuged for 10 min at 1,000 r.p.m. and cultured in Advanced D-MEM/F-12 (DMEM, Invitrogen) plus 100 IU/ml penicillin/streptomycin, 2 mM L-glutamine and 10% FBS (Sigma) in 3% O_2_ and 5% CO_2_ at 37°C. Each cell strain was derived from a separate donor mouse and expanded until enough cells are generated for freezing aliquots. MAFs were thawed at least a week before experiments were conducted. For each experiment, MAFs were seeded at the density of 30000 cells per well of a 12 well plate on sterilized, glass, 19mm (diameter) coverslips, allowed to grow for 24h and then fixed in 2% PFA (young cells) or X-ray irradiated with 10Gy using a PXI X-Rad 225 (RPS Services Ltd) to induce cellular senescence. Medium was changed twice a week. The last medium change was performed at day 20 after senescence induction (IR) and cells were fixed in 2% PFA the next day.

For cytokine measurements, media from the last 24h of culture (before cell fixation) were sent to Eve Technologies for SASP assessment (Mouse Cytokine Array / Chemokine Array 31-Plex (MD31)).

#### Lipid Deprivation Experiments

Under normal conditions, MAFs were kept in Advanced DMEM/F-12 (DMEM, Invitrogen) supplemented with 10% fetal bovine serum (FBS) (Sigma), 100 IU/ml penicillin/streptomycin, and 2 mM L-glutamine. In order to reduce content of lipids in tissue culture media, lipid-deprived FBS (Biowest) was used. Media containing standard FBS (with lipids) was designated “LIPID” and media containing lipid-deprived FBS was designated as “NO LIPID”. Young (non-senescent control) cells were kept for at least 7 days under “NO LIPID” conditions before they were collected or senescence was induced.

#### Mouse Neocortical Astrocytes

Astrocytes were extracted from 16-day-old embryo brains of either sex. At the 16th day of pregnancy, mice were sacrificed and brains of embryos were dissected. Neocortex was isolated and homogenized by pipetting through a fire-polished, FBS-coated Pasteur pipette. Bigger pieces of the neocortex were isolated by sedimentation and supernatant was centrifuged to isolate astrocytes. Astrocyte cultures were seeded at a density of 0.5 × 10^6^ cells/ml on culture dishes that had been coated previously with 15μg/ml poly-l-ornithine overnight and subsequently washed with H_2_O and PBS. Astrocytes were maintained in DMEM/F12 medium supplemented with 5mM HEPES, 33mM glucose, 13mM sodium bicarbonate, 10% fetal bovine serum, 2mM glutamine 100U/ml penicillin, and 100μg/ml streptomycin (all from Invitrogen). Cells were cultured at 37°C in a humidified atmosphere of 5% CO_2_ and 3% O_2_. Induction of senescence and assessment of senescence markers and the ALISE phenotype were performed as for MAFs.

### Method Details

#### Senescent and Young Cells’ Transplantation

Wild-type C57BL/6 mice were obtained from the National Institute on Aging (NIA) and maintained in a pathogen-free facility at 23–24°C under a 12h light, 12h dark regimen with food and water *ad libitum*. Cell transplantation was done as previously described (Xu et al., 2018). Briefly, 18 month old mice were anesthetized using isoflurane and were injected intraperitoneally with 150μl PBS through a 22-G needle containing 10^6^ non-senescent control or senescent mouse preadipocytes or only PBS. Preadipocytes were obtained from inguinal fat from young luciferase-expressing transgenic C57BL/6 mice from the Jackson Laboratory Harbor, (Bar, ME; stock no. 025854). Senescence was induced by 10 Gy cesium radiation. Open field testing was carried out 2 and 6 weeks after transplantation and Rotarod performance was tested 2 and 12 weeks after transplantation.

#### Body Composition

Lean and fat masses of individual mice were determined by quantitative nuclear magnetic resonance using an EchoMRI analyser (Houston, TX) and expressed as a function of body weight. Un-anesthetized animals were placed in a plastic tube that was introduced into the EchoMRI instrument. Body composition, comprising fat mass and lean mass, was determined in approximately 90 seconds per animal.

#### Open Field Testing

Locomotor activity and anxiety-like behavior of mice were assessed in sound-insulated, rectangular activity chambers (Med Associates, St Albans, VT, USA: W×L×D = 27cm×27cm×20cm with continually running fans, infrared lasers, and sensors). Beam breaks were assessed in 2-min bins over 30 min, converted automatically to current mouse location and distance travelled (cm), and recorded on a computer with Med-PC software Version 4.0. Before the test, mice were acclimatized to the room for 1-1.5h before being introduced into the chambers. Mice were habituated for 5 min in the Open Field chamber (without recording) then placed for another 5 min in the home cage. Afterwards, mice were introduced back to the chambers and all mouse movements were recorded for 30 min. Anxiety was quantified by the distance mice travelled in the central 25% of the chamber (zone 1) as a function of the total distance mice travelled and by frequencies of entries into zone 1.

#### Elevated Plus Maze

A grey colored elevated plus maze apparatus was used. Two open arms (25 × 5 cm) and two closed arms (25 × 5 cm) were attached at right angles to a central platform (5 × 5 cm). The apparatus was set 40 cm above the floor. Mice were first acclimatized to the room for 1-1.5h and then placed individually on the central platform with their back to one of the open arms. Before the test, mice were habituated for 1 min to the maze, then placed back in the home cage for 5 min. Mice were tested for 5 min, during which they could freely explore the apparatus. Tracking software (Ethovision) recognized mouse head, central body point, and the base of the tail. Anxiety was quantified by frequency of and time spent during head pokes/dips toward open arms. Higher anxiety is indicated by a lower frequency of movement into open arms and less time spent there.

#### Rotarod

The Rotarod performance test evaluates mouse balance and motor coordination. Mice were brought to the test room a day before testing and habituated overnight. For the baseline tests, mice were first trained on Rotarod (3375-M5; TSE systems) for three consecutive days. Mice were placed (having their back turned towards the experimenter) on the rotating rod of 4.0cm diameter. Mice trained to stay on the rod for 200sec at one constant speed per day, increasing the speed each day from 4rpm, 6rpm, to 8rpm. If a mouse fell during training, it was put back on the rod. For the test on the fourth day, the Rotarod was started at 4rpm and steadily accelerated to 40rpm over a 5 min interval. The speeds at which mice dropped were recorded in 4 consecutive trials. Two and 12 weeks after baseline measurements, mice were tested again, habituating overnight prior to the test day. The average was normalized to the baseline and taken as an indicator of mouse balance and motor coordination.

#### Stone’s T-Maze

A water-motivated version of the Stone’s T-maze was used to measure murine short-term memory. A straight run (for pre-training) or Stone’s T-maze were placed into a steel pan filled with water to a depth of approximately 3 cm so that half the height of the interior walls of the maze were underwater. The ceilings of both the straight run and the maze were covered with clear acrylic to prevent mice from rearing out of the water. These dimensions created a situation that enables the mice to maintain contact with the floor while keeping their heads above water. The mice were placed into a start box and were pushed into the maze using a sliding panel. At the end of the straight run or the maze there was a goal box that contains a ramp to a dry floor, which allows the mice to escape from the water upon successful completion of the straight run or the maze. On day one, mice underwent straight run training to establish the concept that moving forward allows them to escape the water by reaching a water-free goal box. Successful completion of this phase requires the mice to reach the goal box in 10 seconds or faster in 8 out of 10 trials. Mice that did not reach this criterion were excluded from further testing. Maze training commenced the following day. Mice had to complete 9 maze acquisition trials in a single day. All mice per group performed one trial before performing the next one. Runs using between 6 and 8 mice resulted in inter-trial intervals (ITI) of approximately 5-12 minutes. During ITI, mice were placed in a holding cage containing a dry towel that was additionally heated by a red heat lamp. Primary measures of learning and memory were the latency to reach the goal box and the numbers of errors committed. An error was defined as complete entry of the mouse’s head or the whole body into an incorrect path. During the acquisition phase, if any mouse failed to reach the goal box within 5 minutes, the trial was terminated and scored as a failure. Any mouse having 3 failures was removed from further trials. None of the mice was excluded from this study.

#### RT-PCR

Total RNA was extracted from white adipose tissue and brain using Trizol (Life Technologies, Carlsbad, CA) and reverse transcribed to cDNA with an M-MLV Reverse Transcriptase kit (Life Technologies). Real-time PCR was performed in a 7500 Fast Real Time PCR System (Applied Biosystems, Foster City, CA) using TaqMan Fast Universal PCR Master Mix (Life Technologies) and predesigned primers and probes from Applied Biosystems (Assay ID: Mm00494449_m1 [CDKN2A]; Mm04205640_g1 [CDKN1A]). Target gene expression was expressed as 2−ΔΔCT by the comparative CT method and normalized to the expression of TATA-binding protein (TBP) (Assay ID: Mm01277042_m1 [TBP]).

#### Cellular Senescence-Associated Beta-Galactosidase (SA-β-Gal) Activity

On the day of sacrifice, a small piece of adipose tissue was fixed with 2% PFA and 0.5% glutaraldehyde (Sigma) for 15 minutes at room temperature before being incubated overnight in SA-β-Gal solution (150mM NaCl (Sigma), 2mM MgCl_2_ (Sigma), 40mM Citric Acid (Sigma), 12mM NaPO_3_ (Sigma), 400μg/ml X-gal (Thermofisher), 2.1mg/ml potassium hexacyanoferrat(II)trihydrate and 1.65mg/ml Potassium hexacyanoferrat(III)trihydrate (Sigma), pH 6.0) at 37°C overnight. Fat chunks were washed with PBS three times and stored in PBS at 4°C protected from light. Within 3 days, adipose tissue was stained with Hoechst solution (1:5000; Thermofisher), lightly squashed between two 1x3 inch glass slides, and imaged using a light microscope. 10-20 random visual fields were captured at 20x magnification at identical light exposures for all samples. Images were quantified by manual counting of SA-β-Gal positive cells by a blinded assessor and the data were expressed as percent of total DAPI-positive cells.

#### Mass Cytometry/CyTOF in Brain

This technique uniquely combines time-of-flight mass spectrometry with metal-labelling technology to enable detection of up to 40 protein targets per cell. A panel of antibodies based on surface markers, transcription factors, and cytokines (see table below) was designed for brain mass cytometry/cytometry by time of flight (CyTOF). Each antibody was tagged with a rare metal isotope and its function verified by mass cytometry according to the manufacturer’s manual (Multi Metal labelling Kits, Fluidigm, CA). A CyTOF-2 mass cytometer (Fluidigm, South San Francisco, CA) was used for data acquisition. Acquired data were normalized based on normalization beads (Ce140, Eu151, Eu153, Ho165, and Lu175). A single brain hemisphere was dissociated into a single-cell suspension using brain tissue dissociation kits following the manufacture’s instructions. (Adult Brain Dissociation Kit, Miltenyi Biotec, CA). Collected cells were incubated with metal-conjugated antibodies in cell staining buffer (ProductMaxpar Cell Staining Buffer, Fluidigm, CA) and, for testing intracellular proteins including transcription factors and cytokines, fixation and permeabilization was conducted according to the manufacturer's instructions (Transcription Factor Staining Buffer Set, eBioscience, San Diego, CA). CyTOF data were analyzed by Cytobank (Santa Clara, CA).

**Table:** Antibodies Used in CyTOF

Name of an Antigen	Company Producing Antibody	Catalogue Number	Metal Isotopes	Dilution
CD11b (Mac-1)	Fluidigm	3154006B	154Sm	1:200
CD45	Fluidigm	3089005B	89Y	1:400
nestin	Abcam	ab6142	148Nd	1:100
Dcx	Abcam	ab135349	173Yb	1:100
vimentin	Abcam	ab8978	161Dy	1:100
CD133	Biolegend	141202	153Eu	1:100
ACSA-2	Miltenyi Biotec	130099138	142Nd	1:100
Gfap	Ebioscience	14-9892-82	172Yb	1:100
CD31	Fluidigm	3165013B	165Ho	1:100
NeuN	Abcam	ab177487	169Tm	1:50
CD146	Fluidigm	3141016B	141Pr	1:100
CNPase	Abcam	ab53041	151Eu	1:200
OSP	Abcam	ab53041	159Tb	1:200

#### Cytokines

MAFs and astrocytes were plated on 19mm (diameter) coverslips and, at the end of experiments, washed briefly with PBS and fixed for 10 min with 2% paraformaldehyde dissolved in PBS. Cells were permeabilized for 5 min with 0.5% TRITON X-100 diluted in PBS. Cells were incubated with blocking buffer (5% normal goat serum (S-1000, Vector Laboratories) in PBS) for 60 min at room temperature. Plin2 (PROGEN #GP46, 1:250), 53BP1 (Novus Biologicals, #NB100-304, 1:250), and GFAP (Synaptic Systems, #173 004, 1:1000) antibodies were diluted in blocking buffer and applied overnight at 4°C. The next day, cells were washed 3 times with PBS and incubated for 60 min with secondary Alexa Fluor 647, goat, anti-guinea pig antibody (1:1000) for Plin 2 staining; Alexa Fluor 488, goat, anti-rabbit (1:1000) for 53BP1 staining or Alexa Fluor 647, goat, anti-guinea pig (1:1000) for GFAP staining. For quantification of senescence markers, coverslips were washed 3 times in PBS, then mounted in Vectashield, DAPI-containing mounting media. For assessment of lipid accumulation, cells were washed 3 times with PBS before and after DAPI solution (PARTEC), which was added for 30 min at room temperature. 2μl of Nile red solution (Nile red (Sigma N3013) 150μg ml^-1^ in acetone) were added to 1 ml 80% glycerol (in Milli-Q water) and mixed thoroughly. 20μl of Nile Red/glycerol were directly added to each cell sample and mounted on a glass microscope slide. Images were taken immediately after mounting using a Leica DM5500 widefield fluorescence microscope with a 20x objective lens. Area of lipid droplets was quantified using ImageJ (“Analyze particles” tool) in >50 cells in ≥10 images per biological replicate.

#### EdU Experiments

For EdU experiments, HFD and control chow-fed INK-ATTAC mice treated with AP were injected with EdU (Life Technologies; Cat: #E10187) at a dose 123 mg/kg with the final concentration of 6.15mg/mL, dissolved in sterile PBS (pH 7.4, Fisher Scientific), 2 hours before perfusion. There was a 15min interval between mouse injections to allow for time needed to perfuse each mouse. The animals were deeply anesthetized with 90mg/kg ketamine and 10mg/kg of xylazine in sterile PBS prior perfusion. Transcardiac perfusion with PBS was followed by perfusion with 4% paraformaldehyde in PBS chilled on ice. Brains were harvested and postfixed overnight in 4% paraformaldehyde in PBS at 4°C, washed with PBS, and stored at 4°C for vibratome sectioning. Sagittal brain sections of 50μm were cut on a vibratome and collected sequentially in 6 different plate wells, total of 13 sections, 250μm apart in each well, representing 1/6 of the brain hemisphere. Sections were stained free-floating in 12-well plates, with all procedures being performed at room temperature at a volume of 500μl in each well. Sections were initially permeabilized in 4% Triton X-100 (Sigma-Aldrich) in PBS for 1h with 3 subsequent PBS washes. Click reaction was performed for EdU visualization, including 20mM (+)-sodium L-ascorbate (Sigma-Aldrich), 10μM Alexa 555-azide (Life Technologies), and 4mM copper sulfate (Sigma-Aldrich) in PBS. Sections were incubated with gentle shaking for 15min followed by PBS washing. Brain sections were collected and placed on gelatinized glass slides. All preparations were mounted with fluorescent mounting medium (DAKO) and coverslipped.

For assessment of hippocampal neurogenesis, EdU^+^ cells slides were imaged using a Leica DM5500B fluorescence microscope with in depth Z stacking. Cells were manually counted in the basal layer of the dentate gyrus in all 13 sections and multiplied by 6 to obtain an estimate of the number of dividing cells per hemisphere.

For assessment of neurogenesis in the subventricular zone (SVZ), 13 sagittal, 50μm-thick sections were imaged using a Leica DM5500B fluorescence microscope. In depth Z stacking was used (images were captured as stacks separated by 4μm with a 10x objective). Quantity of positive cells was manually counted in the ventral SVZ using ImageJ and the total number of cells was normalized to the number of images taken.

#### Immunostaining and Telomere-Associated Foci (TAF) and Quantification

Paraffin sections were deparaffinized with Histoclear and hydrated in an ethanol gradient followed by water and PBS. Antigen was retrieved by incubation in 0.01M citrate buffer (pH 6.0) at 95°C for 10min. Slides were placed in blocking buffer (1:60 normal goat serum [S-1000, Vector Laboratories] in 0.1% BSA/PBS) for 60min at room temperature. For TAF staining, slides were additionally blocked with Avidin/Biotin (Vector Lab, #SP-2001) for 15min each. Primary antibodies used (table below) were diluted in blocking buffer and applied overnight at 4°C. The next day, slides were washed 3 times with PBS and incubated for 30min with secondary goat, anti-rabbit antibody (1:200; Vector Laboratories #BA-1000) for TAF staining or for 60min with secondary Alexa antibody (table above). For TAF staining, Fluorescein-Avidin in PBS (1:500; #A-2011, Vector Lab) was applied to each sample for 20min. Slides were washed 3 times in PBS, which was followed by FISH for TAF detection. Briefly, tissues were crosslinked with 4% paraformaldehyde for 20min and dehydrated in graded ethanol. Sections were denatured for 10min at 80°C in hybridization buffer (70% formamide (Sigma), 25mM MgCl_2_, 0.1M Tris (pH 7.2), and 5% blocking reagent [Roche]) containing 2.5μg ml^-1^ Cy-3-labelled telomere-specific (CCCTAA) peptide nucleic acid probe (Panagene), followed by hybridization for 2h at room temperature in the dark. Slides were washed twice with 70% formamide in 2×SSC for 15min, followed by washes in 2×SSC and PBS for 10min. Sections were mounted in Vectashield DAPI-containing mounting media and imaged.

A single, 3μm-thick section per mouse was used for TAF staining, while Dcx and Plin2 staining was performed 80μm-apart on three 10μm-thick sections. To quantify periventricular lipid accumulation, 10-30 images in the periventricular region were taken using a DM5500 widefield fluorescence microscope from Leica with a 10x (for frequency of ALISE-positive periventricular glia) or x40 (for assessment of ALISE phenotype of ependymal cells) objective lens. Number of Plin2-positive cells (for frequency of ALISE-positive periventricular glia) or area of Plin2-positive vesicles (for ALISE phenotype of ependymal cells) was assessed using ImageJ software. For assessment of identity of Plin2+ cells, 10μm-thick sections were stained with a combination of antibodies for Iba1 (secondary antibody conjugated with Alexa Fluor 488), Plin2 (secondary antibody conjugated with Alexa Fluor 594), and vimentin (secondary antibody conjugated with Alexa Fluor 647) and quantified for frequency of Plin2+ astrocytes (Iba1-, Vim+) and microglia (Iba1+) in the periventricular region. Separate staining for Plin2 (secondary antibody conjugated with Alexa Fluor 594) and NeuN (secondary antibody conjugated with Alexa Fluor 647) were used to determine frequency of Plin2+ neurons in periventricular region. For TAF quantification, in depth Z stacking was used (images were captured as stacks separated by 0.4 μm with ×63 objective) followed by ImageJ analysis.

Serum levels of the cytokines Eotaxin, G-Csf, Tnf-α, Il-6, Ifn-γ, Il-1α, Il-1β, Il-17, Il-2, Kc/Cxcl1, Mcp-1, M-Csf, Mig, Mip-1α, and Mip-1β were determined using a Multiplexing LASER Bead Assay (Mouse Cytokine Array / Chemokine Array 31-Plex (MD31), Eve Technologies; Canada). Blood was withdrawn from mice by puncturing the sub-mandibular vein on the day of dissection before an animal was sacrificed. 50μL of serum was shipped to Eve Technologies on dry ice. Due to high variability of data, an unbiased elimination of outliers was performed using ROUT’s method (Graphpad 7 Prism). The same panel was used to detect SASP-factors in MAF and 50μl of media were shipped to Eve Technologies on dry ice.

#### Immuno- and Nile Red Staining for MAFs and Astrocytes

**Table:** Antibodies Used in Immunostaining

	Company Producing Primary Antibody and Catalogue Number	Primary Antibody: Origin and Concentration	Secondary Antibody: Origin and Concentration	Tertiary Antibody or Developing System
γ-H2A.X	Cell Signalling, #9718S	Rabbit, 1:250	Anti-rabbit, biotinylated, Goat, 1:200	DSC-fluorescein (Vector Lab)
Perilipin 2 (Plin2)	Synaptic Systems, #GP46	Guinea pig, 1:250	Anti-guinea pig, Alexa 594 or 488, Goat, 1:1000	
Iba1	Abcam, #ab5076	Goat, 1:250	Anti-goat, biotinylated, donkey, 1:1000	
S100β	Synaptic Systems, #287006	Chicken, 1:1000	Anti-guinea pig, Alexa 594 or 647, Goat, 1:1000	
Vimentin	Abcam, #ab24525	Chicken, 1:1000	Anti-guinea pig, Alexa 647, Goat, 1:1000	
Dcx (doublecortin)	Cell Signalling #4604	Rabbit, 1:250	Anti-rabbit, Alexa 594, Goat, 1:1000	
NeuN/FoxO	Abcam ab104224	Mouse, 1:500	Anti-mouse, Alexa 647, Goat, 1:1000	
Gfap	Synaptic systems, #173004	Guinea pig, 1:500	Anti-guinea pig, Alexa 647, Goat, 1:1000	
EdU	Life technologies #E10187		Alex555-azide, 10μm, #A20012	
53BP1	Novus Biologicals, #NB100-304	Rabbit 1:250	Anti-rabbit, Alexa 488, Goat, 1:1000	

#### IHC for GFAP

Tissue distribution of glial fibrillary acidic protein (GFAP) in the brain was assessed by immunohistochemistry using an image analysis workstation after staining with antibodies. The brain sections were pretreated with 0.3% H_2_O_2_ methanol for 1h at room temperature and with normal goat serum for 1h at room temperature. Each specimen was incubated with the primary antibody overnight at 4°C. The primary antibody used in this study and the dilutions were as follows: rabbit anti-cow glial fibrillary acidic protein (GFAP) [1:800, DAKO, Denmark]. Immunohistochemistry was performed using the VECTASTAIN ABC System (Vector Laboratories, Burlingame, CA) with the avidin/biotin peroxidase complex (ABC) method. Negative controls included replacement of the primary antibodies with normal rabbit serum [1:200, DAKO]. Immunoreactivity to rat positive control specimens of the primary antibodies was determined before use.

#### RNA In Situ Hybridization (RNA-ISH)

RNA-ISH was performed after RNAscope protocol from Advanced Cell Diagnostics (ACD). Paraffin sections were deparaffinized with Histoclear, rehydrated in graded ethanol (EtOH), and H_2_O_2_ was applied for 10min at room temperature followed by 2 washes in H_2_O. Sections were placed in hot retrieval reagent and heated for 15min. After washes in H_2_O and 100% EtOH, sections were air dried. Sections were treated with protease plus for 30min at 40°C, washed with H_2_O, and incubated with target probe (p16) for 2h at 40°C. Afterwards, slides were washed with H_2_O followed by incubation with AMP1 (30min at 40°C) and next washed with wash buffer (WB) and AMP2 (15min at 40°C), WB and AMP3 (30min at 40°C), WB and AMP4 (15min at 40°C), WB and AMP5 (30min at RT) and WB, and, finally, AMP6 (15min at RT). Lastly, an RNAscope 2.5 HD Reagent kit-RED was used for chromogenic labelling. After counterstaining with haematoxylin, sections were mounted.

For analysis of cytokines (Il-6 and Cxcl1), sections were co-stained with antibodies for Plin2 and S100β. Briefly, following chromogenic labelling for cytokines, sections were washed 3 times in TBS for 5min each followed by blocking in 0.1%BSA in PBS for 30min at RT. Sections were incubated overnight with primary antibodies at 4°C. Next, sections were washed 3 times in TBS for 5min each followed by secondary antibody incubation for 1h at RT. After 3 TBS washes sections were mounted using ProLong Gold mounting media containing DAPI. Probes used: to detect p16 we used eGFP: 400281, for Il-6: 315891, and for Cxcl1:407721 (all from ADC). For all RNA-ISH experiments data were analyzed by quantifying the % of positive cells (which means each cell containing at least 1 focus was counted as positive).

### Quantification and Statistical Analysis

Data are presented as mean±SEM for all data. All statistical analyses including testing the normality of data distribution were performed using GraphPad Prism 7.01 and a P value <0.05 was considered as significant. The study was designed to compare changes in parameters between lean and obese animals and between obese and obese treated animals independently. All data were assessed for normality using D'Agostino & Pearson normality test (for n>7) or Shapiro-Wilk normality test (for n 7≥n>3). For differences between 2 groups (planned 2-group comparison) t-test were used, data were further tested for equality of variances using F test. For non-normally distributed datasets (p<0.05 in D'Agostino & Pearson or Shapiro-Wilk normality tests), the Mann-Whitney U test was used. For normally distributed datasets, Welch's t-test (if p<0.05 in F test) or Student’s t-test was used. For 2> group comparisons, one-way ANOVA with Tukey’s multiple comparison test was used. For datasets split on two independent factors, 2-way ANOVA was used. Correlations were assessed using Pearson’s (for datasets of normal distribution) or Spearman’s (for datasets of non-normal distribution) rank correlation test. All details regarding statistical tests used for each comparison are in Table S1.

### Data and Software Availability

CyTOF data (selected data, due to space restrictions) have been deposited in Mendeley: http://dx.doi.org/10.17632/w4gnsvjdf6.2. (Full data set will be provided by Yi Zhu: zhu.yi@mayo.edu).

## Supplemental Information

Document S1. Figures S1–S7

Table S1. Statistics Table, Related to Figures 1–7 and S1–S7

Document S2. Article plus Supplemental Information
