# Supplementary material for: Obesity-Induced Cellular Senescence Drives Anxiety and Impairs Neurogenesis
Source: Cell Metab. 2019 May 7;29(5):1061–1077.e8. doi: 10.1016/j.cmet.2018.12.008 (PMC6509403; doi:10.1016/j.cmet.2018.12.008)
Supplement: Document S1. Figures S1–S7 [file mmc1.pdf]

Cell Metabolism, Volume *29*

Supplemental Information

Obesity-Induced Cellular Senescence

Drives Anxiety and Impairs Neurogenesis

Mikolaj Ogrodnik, Yi Zhu, Larissa G.P. Langhi, Tamar Tchkonia, Patrick Krüger, Edward Fielder, Stella Victorelli, Rifqha A. Ruswhandi, Nino Giorgadze, Tamar Pirtskhalava, Oleg Podgorni, Grigori Enikolopov, Kurt O. Johnson, Ming Xu, Christine Inman, Allyson K. Palmer, Marissa Schafer, Moritz Weigl, Yuji Ikeno, Terry C. Burns, João F. Passos, Thomas von Zglinicki, James L. Kirkland, and Diana Jurk

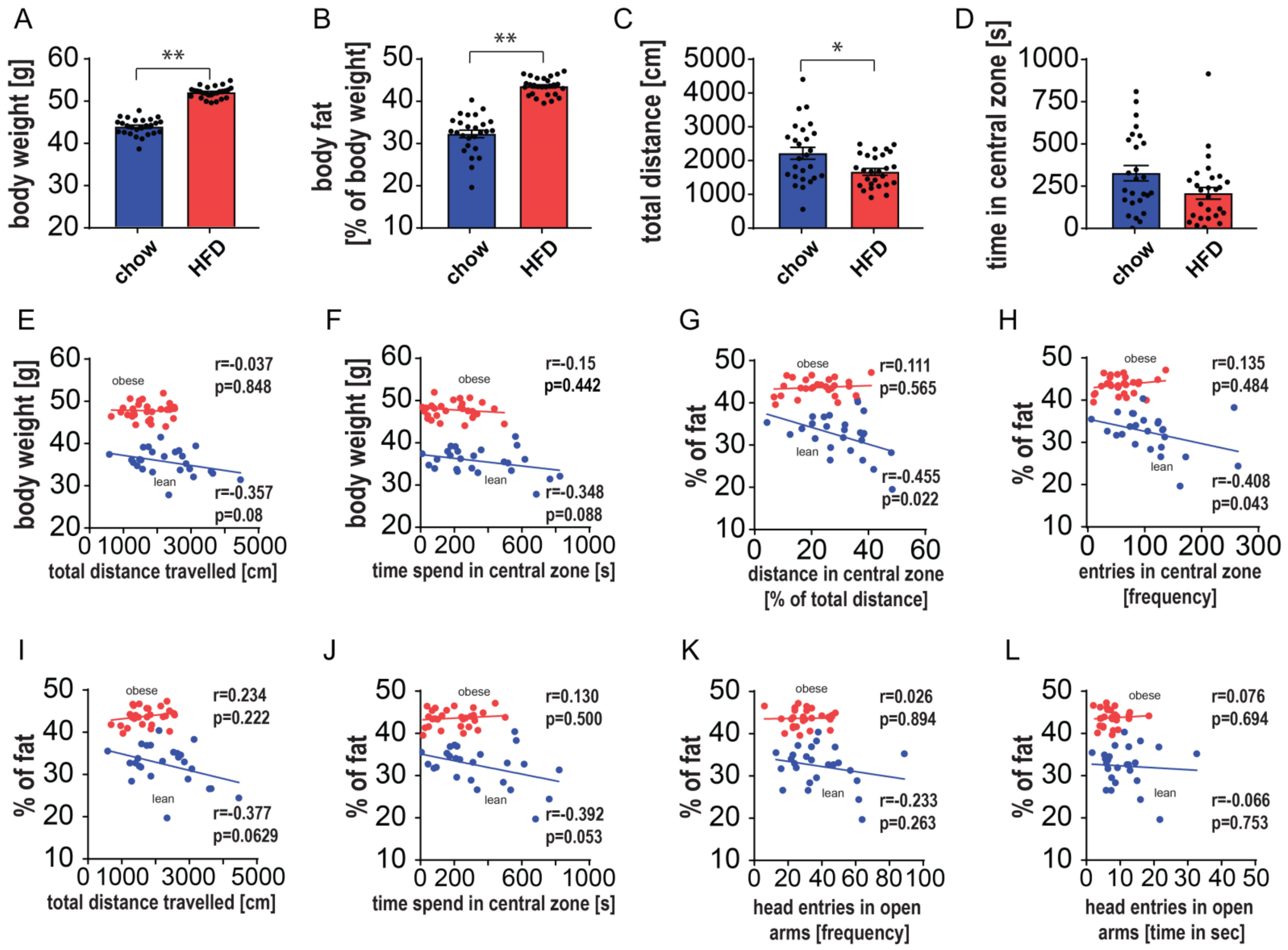

Supplementary Figure S1, related to Figure 1: Further markers of anxiety-like behavior and their correlations with the body mass.

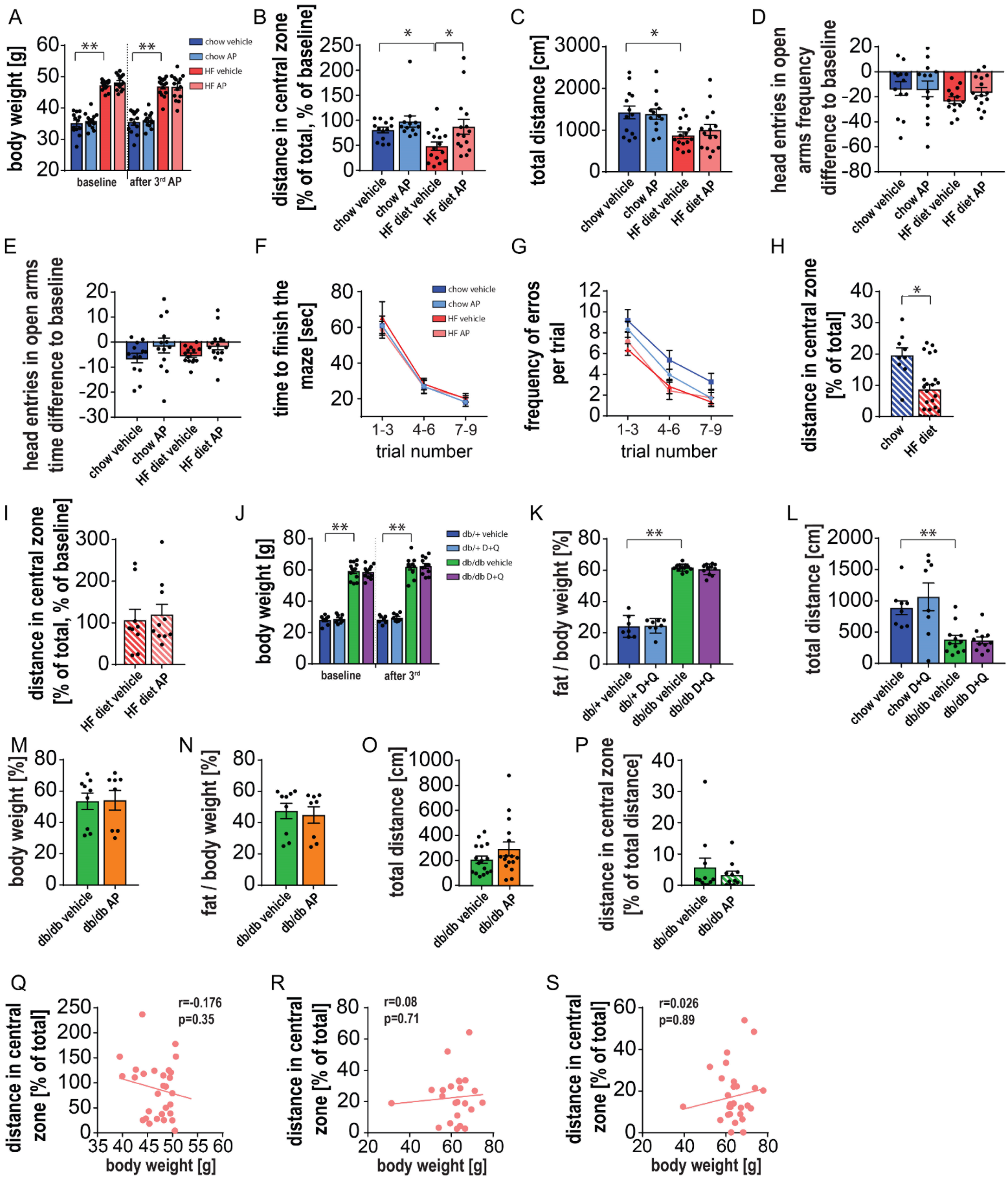

Supplementary Figure S2, related to Figure 2: Additional phenotypic and molecular features of AP20187-treated INK-ATTAC mice.

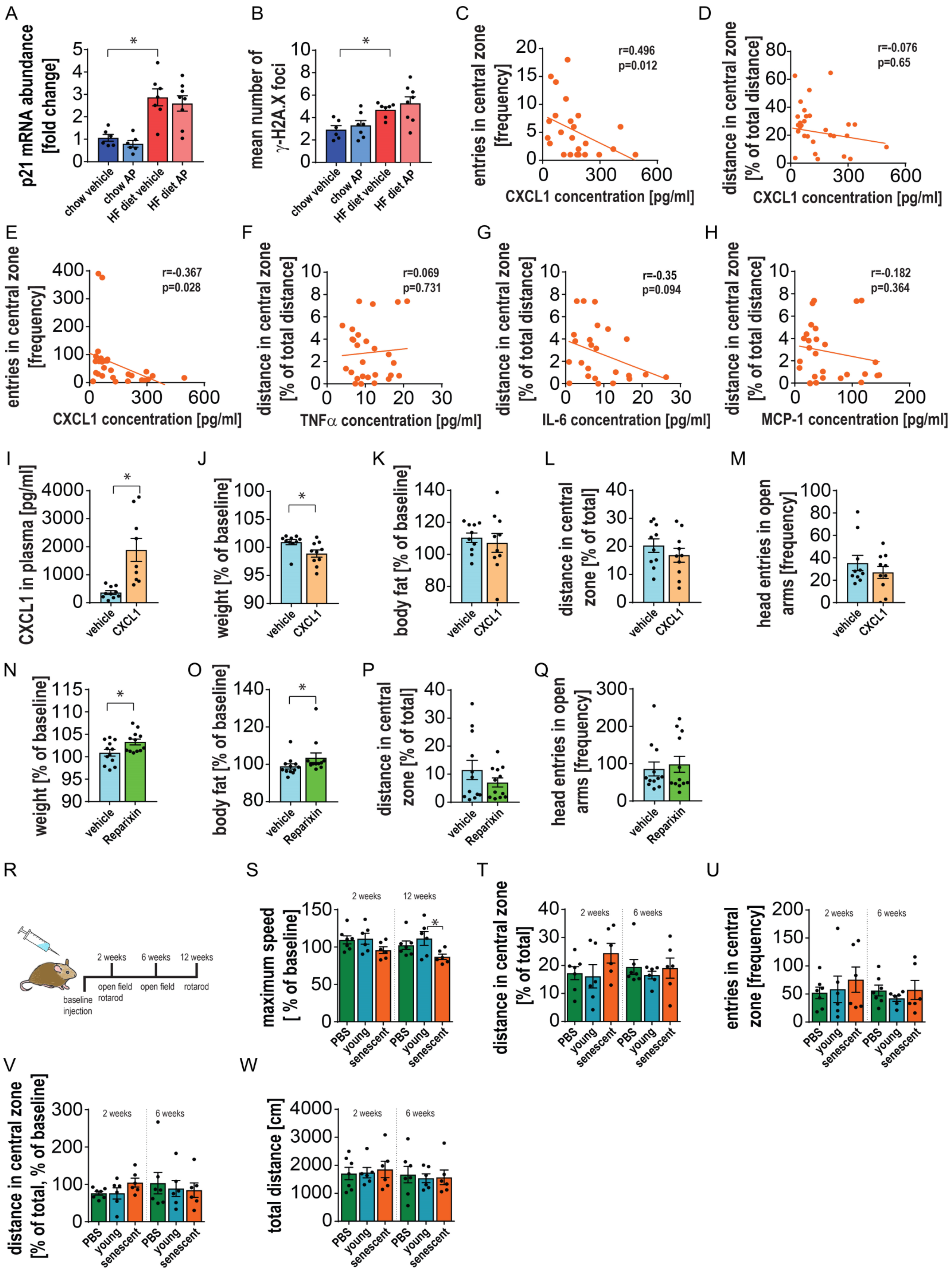

Supplementary Figure S3, related to Figure 3: Influence of systemic factors on anxiety-like behaviour

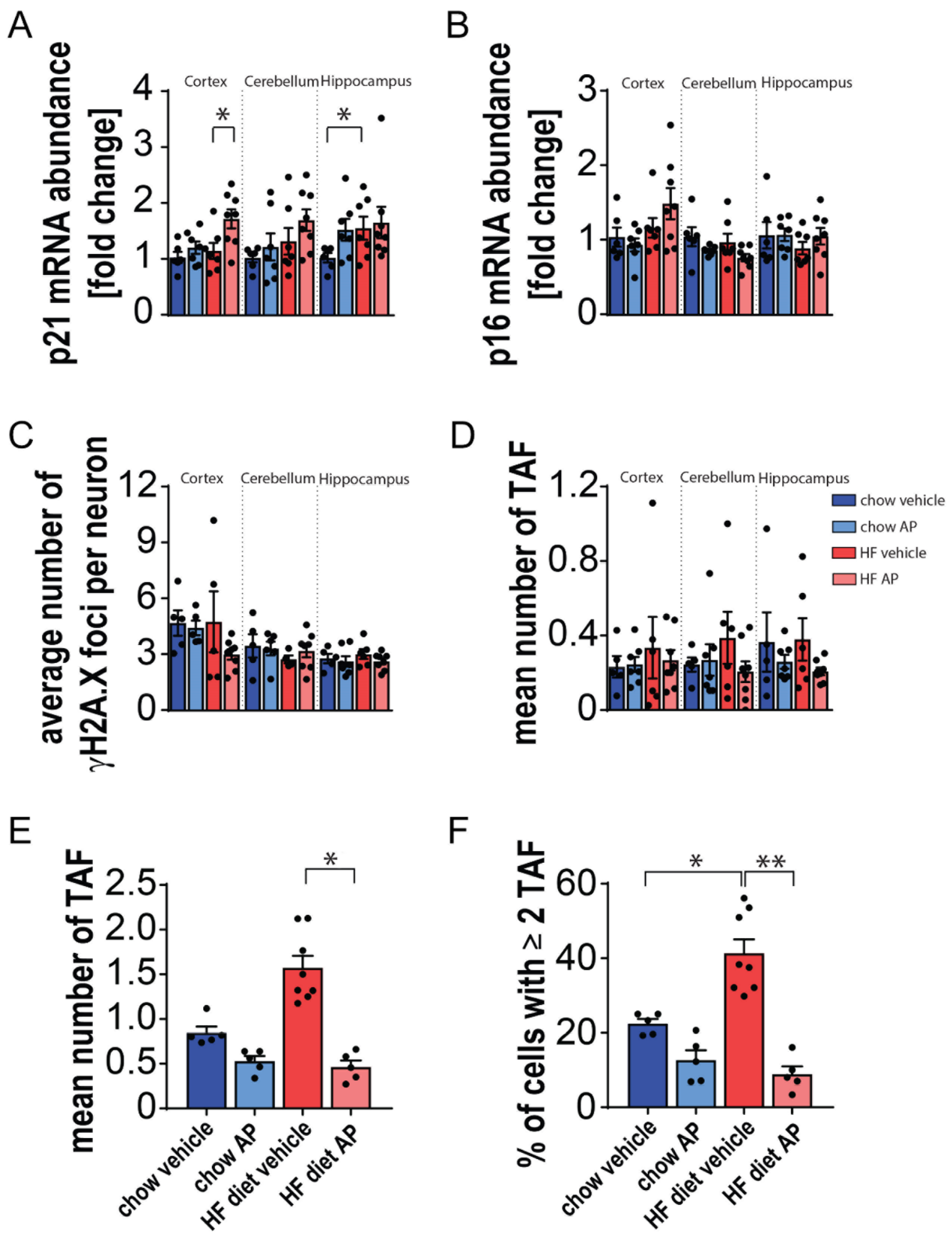

Supplementary Figure S4, related to Figure 4: Levels of senescent markers are not changed in cortex, cerebellum or hippocampus between lean and HFD animals.

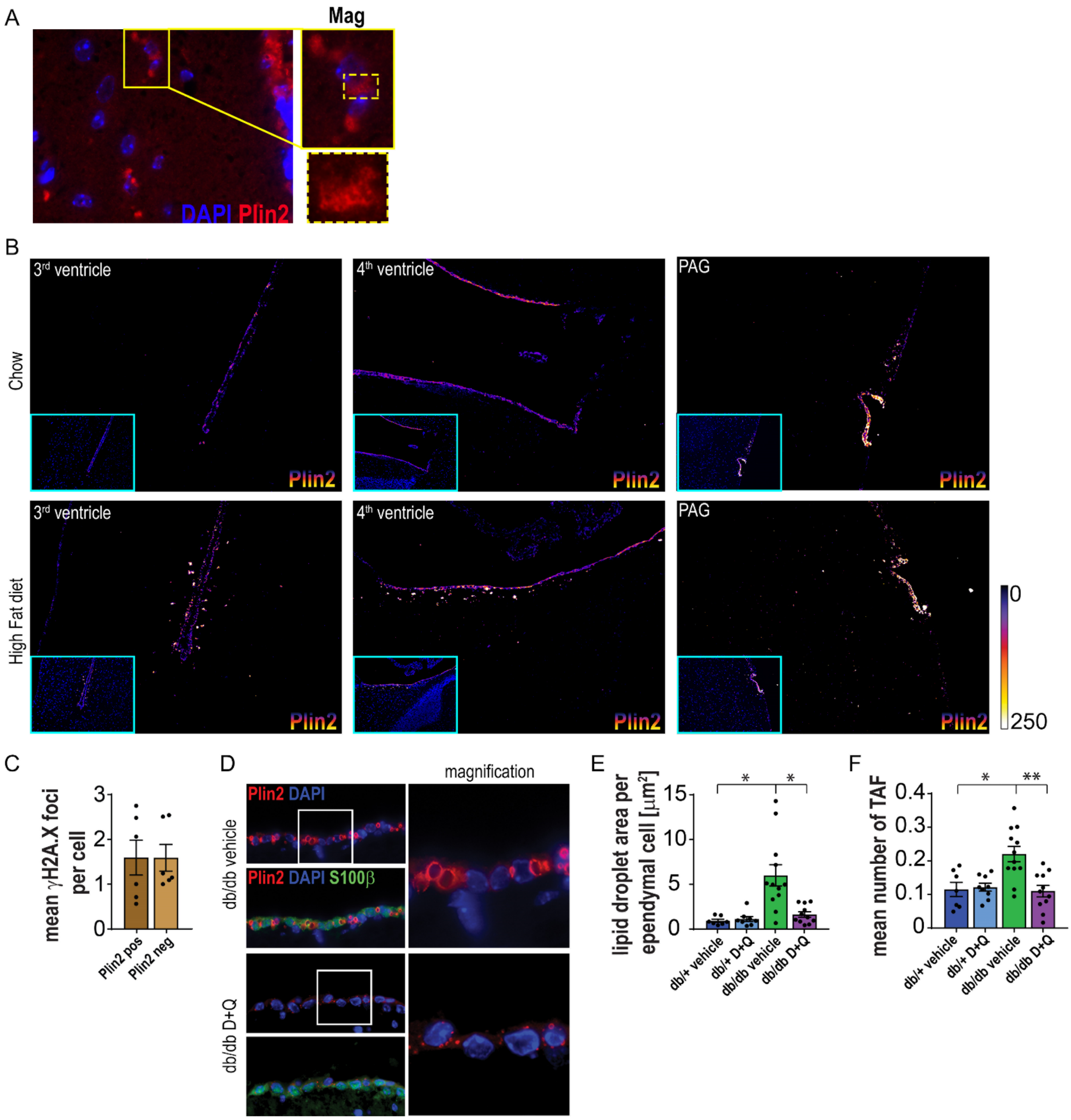

Supplementary Figure S5, related to Figure 5: Assessment of periventricular fat accumulation and markers of senescence in lean and obese mice.

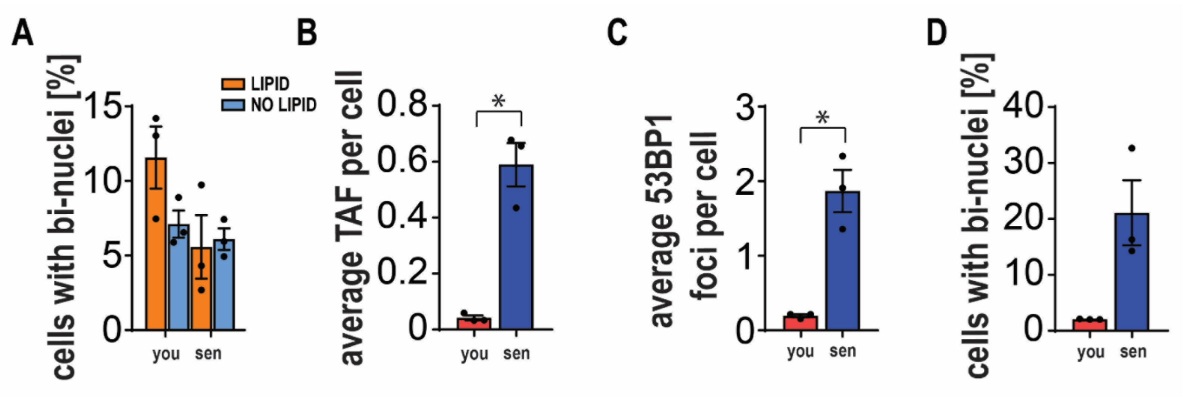

Supplementary Figure S6, related to Figure 6: Analysis of ALISE phenotype in astrocytes

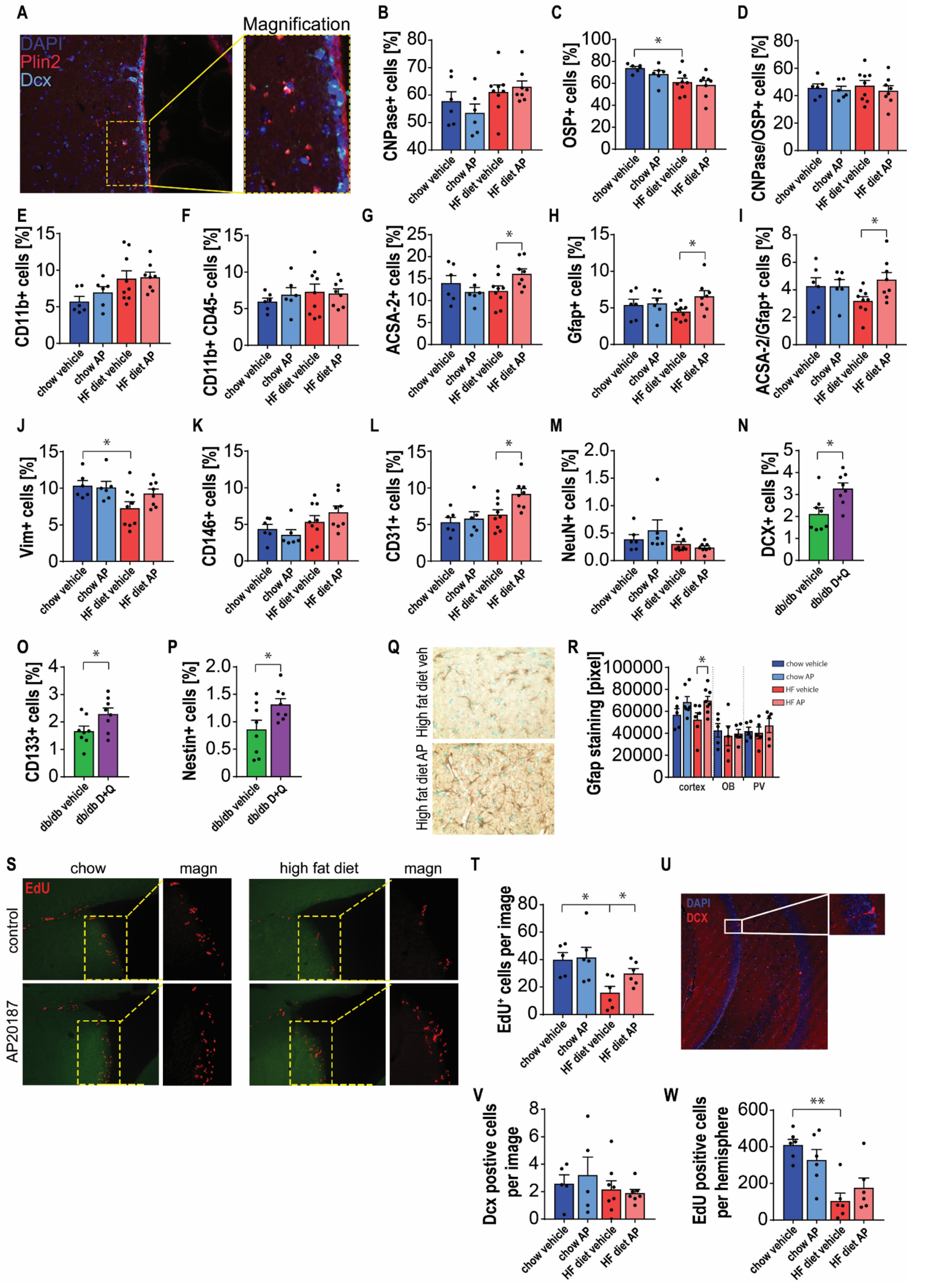

Supplementary Figure S7, related to Figure 7: Associations between adult neurogenesis and periventricular lipid accumulation.

Supplementary Figure S2: Linear regression analysis revealed no significant correlations between anxiety-like behavior markers, body weight, and % body fat in obese mice.

(A) Body mass ($t_{(42.94)}$= 17.66, p<0.0001) and **(B)** normalized body fat in chow- and HFD mice ($t_{(33.37)}$= 11.54, p<0.0001). (**C**) The total distance travelled ($t_{(39.91)}$= 2.754, p=0.0088) and (**D**) total time spent in the central zone of open field (U=273, p=0.0552) in chow- and HFD-fed mice. Linear regression analysis between body mass (**E**), total distance travelled in the open field (r= -0.03721, p= 0.848; r= -0.3569, p= 0.0799), and (**F**) total time spent in the central zone of the open field in lean and obese mice (r= -0.1486, p= 0.4418; r= -0.3479, p= 0.0884). Linear regression analysis between % of body fat and anxiety-like behavior parameters in the open field test: (**G**) normalized distance travelled in the central area (r= 0.1114, p= 0.5651; r= -0.4549, p= 0.0223), (**H**) entries to the central zone (r= 0.135 p= 0.4841; r= -0.4077, p= 0.0431), (**I**) total distance travelled during the test (r= 0.234 p= 0.2218; r= -0.3774, p= 0.0629), and (**J**) time spent in the central area (r= 0.1304, p= 0.5; r= -0.3915, p= 0.053). Body fat of chow- and HFD-fed mice was correlated to parameters of anxiety-like behavior measured in the elevated plus maze test: (**K**) frequency of head pokes into the open arms (r= 0.02577, p= 0.8944; r= -0.2329, p= 0.2625) and (**L**) time of head spend in open arms (r= 0.07626, p= 0.6942; r= -0.06627, p= 0.753).

Data are from: n=26-30 mice per group, Mean ±SEM plotted. * P≤0.05 and ** P≤0.001.

Supplementary Figure S2: Additional phenotypic and molecular features of AP20187-treated INK-ATTAC mice.

(A) Body mass measurements in chow- and HFD-fed mice before and after the last treatment with AP20187 (AP) shows no change in body weight over time (left $t_{(16.16)}$= 11.58, p<0.0001; $t_{(28)}$= 1.242, p= 0.2245) (right $t_{(26)}$= 9.379, p<0.0001; $t_{(28)}$= 0.04832, p=0.9618). Additional parameters from the open field test: **(B)** distance in the middle area as a function of the total distance travelled and to the baseline (measurements before the treatment) ($t_{(26)}$= 3.163, p=0.0039; U= 59, p= 0.0264) and **(C)** total distance travelled ($t_{(18.3)}$= 3.152, p= 0.0054; $t_{(28)}$= 0.8227, p= 0.4176). Normalized to baseline parameters of elevated plus maze testing: **(D)** head pokes toward the open arms ($t_{(17.73)}$= 1.528, p= 0.1442; $t_{(27)}$= 1.615, p= 0.118) and **(E)** time spent with the head in the open area of the maze ($t_{(17.04)}$= 0.5744, p= 0.5732; $t_{(20.69)}$= 1.976, p= 0.0617). **(F, G)** Short-term memory was not affected by obesity or AP treatment as determined by lack of substantial changes in Stone's maze parameters: **(F)** time needed to finish the maze (p=0.4713) and **(G)** frequency of errors (p=0.685). C57Bl6 wild-type mice were used to test off-target effects of AP. **(H)** Before

treatment, HFD mice had anxiety-like behaviour (measured by distance travelled in the centre of the open field box) ($t_{(15.93)}$= 3.756, p= 0.0017). **(I)** Treatment of HFD C57Bl6 mice with AP showed no difference in behavior between treated and un-treated mice (U= 42, p= 0.8421). *Db/db* and heterozygous lean (db/+) mice were given 2 months of D+Q treatment for 5 days every 2 weeks starting from the age of 4 months. Db/db mice did not show changes in **(J)** body mass (left $t_{(18)}$= 14.59, p<0.0001; $t_{(21)}$= 0.3297, p= 0.7449) (right $t_{(13.78)}$= 15.58, p<0.0001; $t_{(20)}$= 0.214, p =0.8327) or **(K)** body fat within the groups over the course of treatment ($t_{(6.713)}$= 13.85, p<0.0001; $t_{(21)}$= 0.9251, p= 0.3654). **(L)** Total distance travelled by *db/db* and *db/+* mice in the open field test was not affected by D+Q treatment ($t_{(18)}$= 4.173, p=0.0006; $t_{(21)}$= 0.1439, p= 0.8869). 3-month old INK-ATTAC; INK-ATTAC:*db/db* mice were randomly sorted to AP or vehicle groups and treated for 2 months. No changes in **(M)** body mass ($t_{(15)}$= 0.08256, p= 0.9353) or **(N)** body composition ($t_{(15)}$= 0.3487, p= 0.7322) were observed in AP treated mice. **(O)** Total distance travelled during 30min long open field testing was not affected by AP treatment (U= 97, p= 0.2542). *Db/db* mice were tested for off-target effects of AP. **(P)** Treatment showed no difference in behavior between treated and un-treated mice (U= 56, p= 0.7955). **(Q-S)** Linear regression analysis between body weight and anxiety-like behavior, measured as distance travelled in the centre of the open field box, showed no association between the two in **(Q)** HFD INK-ATTAC mice treated with and without AP (r= -0.1755, p= 0.3535), **(R)** in *db/db* mice treated with and without D+Q (r= 0.07974, p= 0.7176), and in **(S)** INK-ATTAC:*db/db* mice treated with and without AP20187 (r= 0.02621, p= 0.8887).

Data are from n=13-15 mice per group for graphs A-E, n=9-24 mice per group for F-G, n=7-12 mice per group for H, n= 9-10 mice per group for graphs I, n=6-8 mice per group for graphs J-L, n=8-9 mice per group for graphs M-N, n=16 mice per group for the graph O, n= 11 mice per group for the graph P, n= 30-31 mice per group for graphs Q and S and n=23 mice per group for the graph R. Mean ± SEM plotted. * P≤0.05 and ** P≤0.001.

Supplementary Figure S3: Influence of systemic factors on anxiety-like behavior.

Quantification of **(A)** p21 by PCR ($t_{(7.81)}$= 4.419, p=0.0024; $t_{(12.61)}$= 0.5318, p= 0.6041) and **(B)** the number of DNA damage foci (γ-H2A.X) by IF-staining ($t_{(11)}$= 4.224, p=0.0014; $t_{(9.293)}$= 0.8931, p= 0.3943) in perigonadal adipose tissue shows increased values in HFD-fed animals and but no substantial change after treatment with AP20187. Correlations between anxiety-like phenotype markers and Cxcl-1 in blood plasma of HFD- and chow-fed animals **(C)** (r= -0.4968, p= 0.0115) in EPM, **(D)** (r= -0.0761, p= 0.6544) OF and in *db/db* and

$db/db^{+/-}$ animals **(E)** (r= -0.3672, p= 0.0276) EPM. Correlations between **(F)** Tnf-α (r= 0.06926, p= 0.7314), **(G)** Il-6 (r= -0.35, p= 0.0936), and **(H)** Mcp-1 (r= -0.1815, p= 0.3648) in blood plasma of HFD- and chow-fed diet animals and the distance travelled in the central zone of the open field box showed no significant differences. To test the effect of increased or decreased cytokine levels in the blood stream mice were injected with Cxcl-1 or treated with the Cxcr1 inhibitor, Reparixin. **(I)** Cxcl-1 is significantly increased in blood plasma of C57Bl6 mice injected with Cxcl-1 compared to vehicle-treated mice ($t_{(17)}$= 3.842, p= 0.0013). Mice injected with Cxcl-1 **(J)** lost weight (U= 17, p= 0.0115) but **(K)** did not experience changes in body fat ($t_{(18)}$= 0.4908, p= 0.6295). **(L)** Distance travelled in the central area of the open field box ($t_{(18)}$= 0.9984, p= 0.3313) and **(M)** head entries into the open arms of the EPM (U= 45, p= 0.7391) were not substantially different between Cxcl-1- and vehicle-treated mice. HFD mice treated with Reparixin or vehicle showed **(N)** an increase in body mass ($t_{(18)}$= 0.6908, p= 0.0239), but **(O)** did not show alterations in body fat (U= 30, p= 0.0145). No difference in **(P)** the open field ($t_{(15.68)}$= 1.173, p= 0.2582) and **(Q)** EPM (U= 72, p= >0.9999) tests were observed. **(R)** Scheme showing experimental setup for transplantation experiments. C57Bl6 mice were divided into 3 groups, injected either with PBS, young (non-senescent) or senescent mouse preadipocytes and tested for anxiety-like behavior (OF) and frailty (Rotarod). **(S)** Assessment of Rotarod showed significantly decreased performance in mice injected with senescent cells 12, but not 2, weeks after injection (left $t_{(10)}$= 1.795, p= 0.1029; right $t_{(10)}$= 2.477, p= 0.0327). All mice had no anxiety-like phenotype 2 and 6 weeks after cell transplantation, when **(T)** distance travelled(left $t_{(10)}$= 1.518, p= 0.16; right $t_{(10)}$= 0.8496, p= 0.5315), **(U)** entries (left $t_{(10)}$= 0.5262, p= 0.6102; right $t_{(5.884)}$= 0.8496, p= 0.4288)**,** **(V)** normalized distance travelled in the central area (left $t_{(10)}$= 1.493, p= 0.1662; right $t_{(10)}$= 0.1542, p= 0.8805), and **(W)** total distance travelled (left $t_{(10)}$= 0.3265, p= 0.7508; right $t_{(10)}$= 0.1096, p= 0.9149) in the open field box were measured.

Data are from n= 6-8 mice per group for graphs A-B, mice per group n= 27 for graphs C and F-H, mice per group n= 36 for graphs D-E, n=10 mice per group for graphs I-M, n=12 mice per group for graphs N-Q and n=6-7 mice per group for the graphs S-W. Mean ± SEM plotted. * P≤0.05 and ** P≤0.001.

Supplementary Figure S4: Levels of senescence markers are not changed in cortex, cerebellum, or hippocampus of HFD compared to lean animals.

Analysis of **(A)** p21 (Cortex $t_{(11)}$= 0.6522, p= 0.5277; $t_{(13)}$= 2.54, p= 0.0246) (Cerebellum $t_{(7.313)}$= 1.173, p= 0.2777; $t_{(13)}$= 1.247, p= 0.2344) (Hippocampus $t_{(7.905)}$= 2.359, p= 0.0464; U= 27, p= 0.9551) and **(B)** p16

(Cortex U= 15, p= 0.4452; U= 21, p= 0.4634) (Cerebellum $t_{(11)}$= 0.4379, p= 0.6699; $t_{(13)}$= 1.741, p= 0.1053) ($t_{(11)}$= 0.9306, p= 0.372; Hippocampus $t_{(13)}$= 1.113, p= 0.2857) by RT-PCR in different brain regions (cortex, cerebellum, and hippocampus) showed no significant difference among groups. Quantification of **(C)** γH2A.X foci (Cortex $t_{(8)}$= 0.03504, p= 0.9729; $t_{(4.235)}$= 1.056, p= 0.3476) (Cerebellum $t_{(7)}$= 0.9903, p= 0.355; $t_{(10)}$= 0.8641, p= 0.4078) (Hippocampus $t_{(9)}$= 0.6328, p= 0.5426; $t_{(12)}$= 1.342, p= 0.2044) and **(D)** telomere associated damage foci (TAF) (Cortex U= 14, p= 0.9307; U= 21, p= 0.7296) (Cerebellum $t_{(5.722)}$= 0.9935, p= 0.3606; $t_{(12)}$= 1.346, p= 0.2033) (Hippocampus $t_{(9)}$= 0.07184, p= 0.9443; $t_{(5.317)}$= 1.48, p= 0.1956) in neurons in different brain areas showed no significant difference. **(E)** Mean number of TAF ($t_{(11)}$= 0.9522, p= 0.3614; $t_{(11)}$= 3.588, p= 0.0043) and **(F)** % of NeuN-pos cells with 2 or more TAF ($t_{(8.475)}$= 4.773, p= 0.0012; $t_{(11)}$= 6.381, p<0.0001) was increased in HFD INK-ATTAC mice and significantly reduced after AP20187 treatment in the hypothalamus in close proximity to the 3rd ventricle.

Data are from n=5-8 mice per group for graphs A-D; n= 5 for E, F. Mean ± SEM plotted. * P≤0.05 and ** P≤0.001.

Supplementary Figure S5: Assessment of periventricular fat accumulation and markers of senescence in lean and obese mice.

(A) Lipid droplets in periventricular cells visualized by Perilipin 2 (Plin2) staining. The panel on the top right shows a magnified cell and the panel on the bottom right shows magnified lipid droplets visualized by Plin2 staining. **(B)** Plin2 staining shows accumulation of lipid droplets in cells in close proximity to the 3rd (left panel), the 4th ventricle (middle panel), and periaqueductal grey matter (PAG) (right panel). Bottom left images show merged images of Plin2 and DAPI staining. **(C)** γ-H2A.X foci were quantified in $Plin2^+$ and $Plin2^-$, non-neuronal cells in the lateral ventricle (U= 14.5, p= 0.619). **(D)** Images showing the ependymal layer in the LV of *db/db* vehicle (top panel) and *db/db* D+Q-treated mice stained with Plin2 in red and S100β in green. Areas in white rectangles are magnified on the right and show reduction in size of Plin2 lipid droplets in D+Q-treated animals. **(E)** Quantification of the area containing lipid droplets ($Plin2^+$) in the ependymal layer of *db/db* and $db/db^{+/-}$ mice with or without D+Q treatment ($t_{(11.5)}$= 4.234, p= 0.0013; $t_{(12.45)}$= 3.566, p= 0.0037). **(F)** Quantification of frequencies TAF-positive cells in the ependymal layer of *db/db* and *db/+* and vehicle- or D+Q-treated mice ($t_{(17)}$= 3.101, p= 0.0065; $t_{(21)}$= 3.842, p= 0.0009).

Data are from n= 6 mice per group for graph C, n=7-12 mice per group for graph E, F. Mean ± SEM plotted. * P≤0.05 and ** P≤0.001.

Supplementary Figure S6: Analysis of the ALISE phenotype in astrocytes

(A) Suppression of the ALISE phenotype by culturing cells in lipid free media does not change bi-nuclearity in MAFs (p=0.1091; p=0.9953). In astrocytes, **(B)** the average number of TAF ($t_{(4)}$= 7.019, p= 0.0022), **(C)** average number of 53BP1 foci ($t_{(2.021)}$= 5.887, p= 0.027), and **(D)** bi-nuclearity ($t_{(2)}$= 3.278, p= 0.0818) increases significantly after induction of senescence. Senescence was induced by X-ray irradiation (10Gy) and established within 14-21 days post-irradiation. Data are from n=3 mice. Mean ±SEM plotted. * P≤0.05 and ** P≤0.001.

Supplementary Figure S7: Association between adult neurogenesis and periventricular lipid accumulation.

(A) Cells displaying perilipin 2 (Plin2)-positive lipid droplets were found in close proximity to doublecortin (Dcx)-positive cells. Yellow box marks magnified region shown on the right.

Characterization by Cytometry by Time of Flight (CyTOF) of different cell types in the brain of lean and obese INK-ATTAC mice with or without AP treatment. Quantification of markers of oligodentrocytes: **(B)** 2',3'-cyclic-nucleotide 3'-phosphodiesterase (CNPase) ($t_{(13)}$= 0.7929, p= 0.4421; U= 35, p= 0.9626), **(C)** oligodendrocyte specific protein (OSP) ($t_{(10.83)}$= 3.331, p= 0.0068; $t_{(15)}$= 0.4891, p= 0.6318), **(D)** double positive cells for CNPase and OSP ($t_{(13)}$= 0.3173, p= 0.7561; $t_{(15)}$= 0.6897, p= 0.5009); markers of microglia: **(E)** CD11b (U= 10, p= 0.0496; $t_{(15)}$= 0.1531, p= 0.8803) and **(F)** $CD45^{-}/CD11b^{+}$ ($t_{(11.04)}$= 1.122, p= 0.2855; $t_{(15)}$= 0.1777, p= 0.8614); markers of astrocytes: **(G)** astrocyte cell surface antigen-2 (ACSA-2) ($t_{(13)}$= 0.8721, p= 0.3989; $t_{(15)}$= 2.375, p= 0.0313), **(H)** glial fibrillary acidic protein (Gfap) ($t_{(13)}$= 1.197, p= 0.2528; $t_{(15)}$= 2.639, p= 0.0186), and **(I)** double-positive for ACSA-2 and Gfap ($t_{(13)}$= 1.701, p= 0.1128; $t_{(15)}$= 2.59, p= 0.0205); **(J)** marker of astrocytes, epithelial cells, pericytes, and ependymal cells: vimentin (Vim) ($t_{(13)}$= 2.458, p= 0.0288; $t_{(15)}$= 1.804, p= 0.0913); markers of endothelial cells and pericytes: **(K)** CD146 ($t_{(13)}$= 0.8256, p= 0.4239; $t_{(15)}$= 1.084, p= 0.2955) and **(L)** CD31 ($t_{(13)}$= 1.054, p= 0.3109; $t_{(15)}$= 2.816, p= 0.013); and a **(M)** marker of neurons, NeuN $t_{(13)}$= 0.9677, p= 0.3508; ($t_{(15)}$= 1.129, p= 0.2766). One hemisphere of each *db/db* vehicle- or D+Q-treated mouse brain was dissociated into a single-cell suspension and labelled with metal-conjugated antibodies and processed for CyTOF. Frequencies of cells expressing markers **(N)** doublecortin (Dcx) ($t_{(14)}$= 2.994, p= 0.0097), **(O)** CD133 ($t_{(14)}$= 2.187, p= 0.0462), and **(P)** Nestin ($t_{(14)}$= 2.3, p= 0.0374) were quantified. **(Q)** GFAP staining (blue is DAPI) in the cerebral cortex and **(R)** its quantification (in the indicated areas) (Cortex $t_{(9)}$= 0.5331, p= 0.6069; $t_{(12)}$= 2.423, p= 0.0322) (OB $t_{(8)}$=

0.4559, p= 0.6606; $t_{(8)}$= 0.2026, p= 0.8445) (PV $t_{(8)}$= 0.2331, p= 0.8216; $t_{(8)}$= 0.7942, p= 0.45). **(S)** Representative images of EdU staining in the preventricular area of chow- and HFD-fed mice treated with vehicle or AP20187. Yellow boxes show magnified regions. **(T)** Quantitative analysis of EdU-positive cells per image in lean and obese INK-ATTAC mice with or without AP treatment ($t_{(9)}$= 3.443, p= 0.0074; $t_{(10)}$= 2.412, p= 0.0365). **(U)** A representative image of immunofluorescent staining for DCX in the dentate gyrus (DG) of the hippocampus. **(V)** Frequencies of DCX-positive cells in the DG ($t_{(11)}$= 0.4418, p= 0.6672; $t_{(9.552)}$= 0.4072, p= 0.6928) and **(W)** total amount of EdU-positive cells ($t_{(10)}$= 5.792, p= 0.0002; $t_{(10)}$= 1.05, p= 0.3186) in the hippocampus per hemisphere in lean and HFD INK-ATTAC animals with and without AP treatment.

Data are from n=6-9 mice per group for graphs B-M, n=8 mice per group for graphs N-P, n=5-8 mice per group for the graph R, n=3 mice per group for the graph T, n=5-8 mice per group for the graph V, n=6 mice per group for the graph W. Mean +/- SEM plotted. * P≤0.05 and ** P≤0.001.
